# A Soft Robotic Model for Simulating Heart Valve Disease and Cardiac Interventions

**DOI:** 10.1002/advs.202516667

**Published:** 2026-02-21

**Authors:** James Davies, Emanuele Nicotra, Kefan Zhu, Chi Cong Nguyen, Bibhu Sharma, Adrienne Ji, Phuoc Thien Phan, Jingjing Wan, Patrick Pruscino, Hermione Truong, Jelena Rnjak‐Kovacina, Hoang‐Phuong Phan, Christopher Hayward, Nigel Hamilton Lovell, Thanh Nho Do

**Affiliations:** ^1^ School of Biomedical Engineering, Faculty of Engineering University of New South Wales Sydney Sydney New South Wales Australia; ^2^ School of Mechanical and Manufacturing Engineering, Faculty of Engineering University of New South Wales Sydney Sydney New South Wales Australia; ^3^ Department of Cardiology St Vincent's Hospital Sydney New South Wales Australia; ^4^ St Vincent's Clinical School, Faculty of Medicine University of New South Wales Sydney Sydney New South Wales Australia; ^5^ Tyree Institute of Health Engineering (IHealthE) University of New South Wales Sydney Sydney New South Wales Australia

**Keywords:** cardiac catheter, cardiac intervention, heart simulator, heart valve diseases, soft robotics

## Abstract

The heart is a dynamic, multisystem organ whose motion is dictated by a complex but organized muscular architecture. Each component is vital to its performance, with the valves being especially critical for ensuring pumping efficiency, yet they are among the most common sites of pathology. Treatment of heart disease is being delivered via minimally invasive procedures to improve patient outcomes and experience. The development of new surgical tools and procedures would greatly benefit from a stable, controllable, and biomimetic simulation platform that accurately replicates the heart's biomechanics and hemodynamics, yet no such system exists. This work introduces a fully synthetic, soft robotic left heart simulator capable of replicating the complex motions of native heart. Its advanced fabrication process is programmable, enabling both patient‐ and disease‐specific cardiac geometries, structures, and valves. The simulator is compatible with clinical echocardiographic imaging, enabling identification of various valvular pathologies based on clinical diagnostic criteria. Its utility is further demonstrated through the evaluation of a new soft robotic cardiac catheter with in‐situ contact force sensing and feedforward, data‐driven control system. With controllable artificial musculature and customizable internal cardiac structures and geometry, the simulator offers a robust platform for investigating cardiac disease and assessing emerging therapeutic technologies.

## Graphical abstract

This paper introduces a fully synthetic fabrication methodology for a soft robotic, in‐vitro model of the left‐heart, complete with a functioning mitral valve apparatus. With a view towards patient‐specific modeling, we demonstrate physiological flow and pressure waveforms, tunable mitral valve function with clinical imaging compatibility, and its use in the assessment of a novel intracardiac catheter.

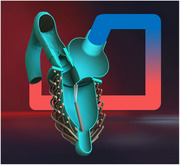

## 1 Introduction

**FIGURE 1:**
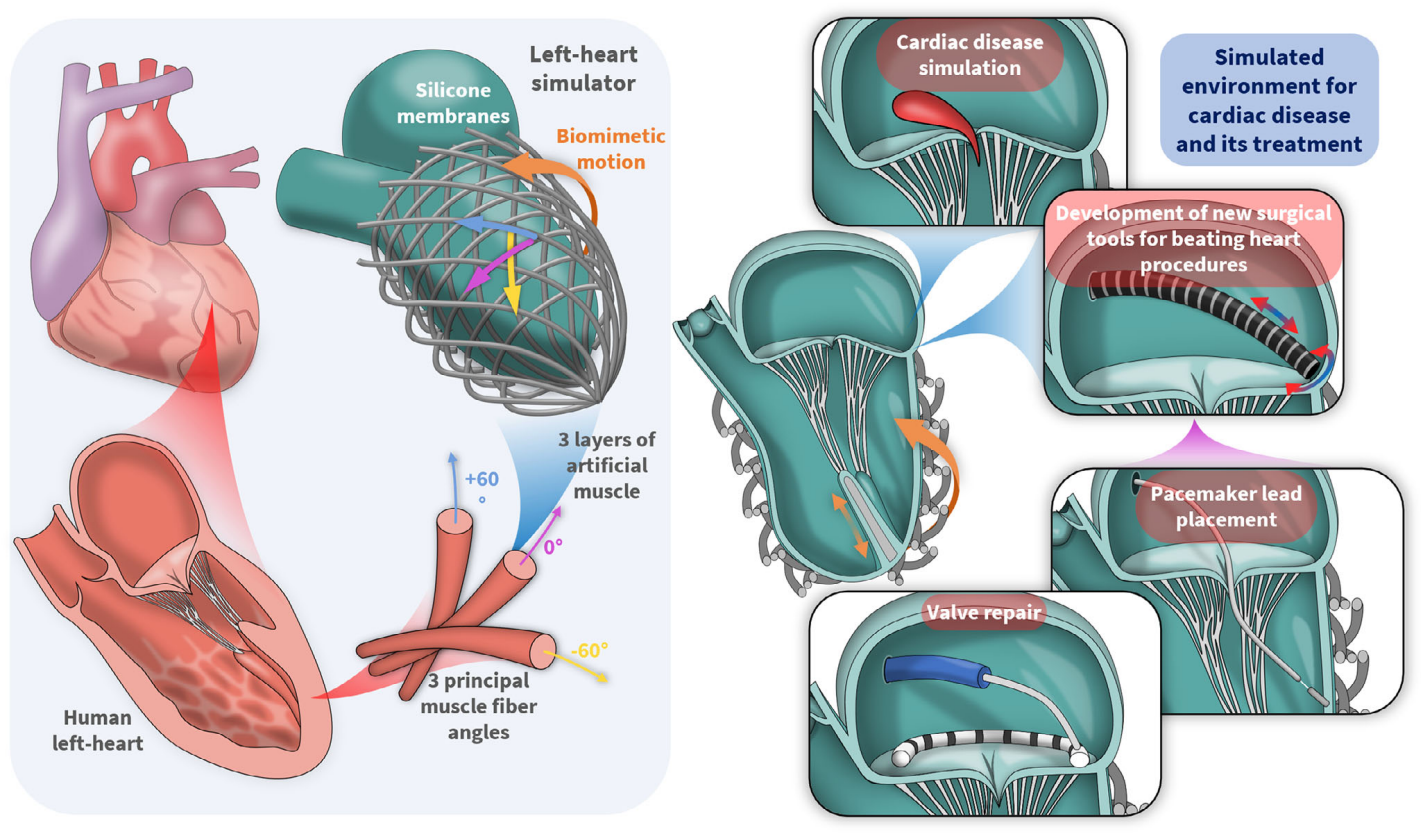
Biomimetic design of the left heart simulator and its application in the study of cardiac disease and the design and development of next‐generation cardiac tools and therapeutic interventions.

The human heart is an incredible piece of evolutionary ‘engineering’. It is an intricate, interdependent, and multicomponent structure. Changes to any component of the heart can significantly affect the efficiency of the entire organ. The musculature of the heart is spatially encoded by complex electrical conduction pathways, which trigger contraction‐relaxation patterns optimized for pumping efficiency. One of the most important elements of the heart is its valves. Without their function, separating the heart's chambers of the heart, its musculature cannot produce unidirectional flow, nor generate the pressure necessary to perfuse the tissues of the body. As with any complex engineered system, individual components of the heart can become dysfunctional over time. Among heart pathologies, valvular heart disease is a significant burden on global healthcare systems, with approximately 83.6 million cases reported globally in 2021 [1]. Valvular heart disease can take multiple forms, but perhaps the most common is mitral regurgitation (MR), characterized by leakage of the mitral valve (MV) during systole, resulting in reduced cardiac output and elevated back‐pressure into the pulmonary circulation (Figure 1).

In recent years, there has been a shift towards minimally invasive treatment options for such a disease. Compared to traditional approaches, transcatheter procedures are less traumatic and offer much faster patient recovery times, even same‐day discharge, naturally reducing costs to both patients and healthcare systems [2, 3, 4, 5]. However, operating within the beating heart is difficult and requires devices with precise and intuitive design and control. While transcatheter aortic valve replacement (TAVR) is well established and is performed in a relatively stable region of the heart, transcatheter techniques for the mitral valve, such as transcatheter edge‐to‐edge repair (TEER) are far more complex. This can be attributed to the dynamic motion of the mitral valve leaflets, chordae tendinea, and papillary muscles, all of which are critical for proper mitral valve function.

Cardiac arrhythmias also represent a major clinical issue, accounting for 59 million cases worldwide in 2019 [6]. To treat abnormal heart rhythms (arrhythmias) when the heart's natural electrical system is impaired, cardiac ablation and pacemaker leads are utilized. Transcatheter tools are used to perform ablation and pacemaker lead placement in both the atria and ventricles while the heart is beating. However, the dynamics of cardiac wall motion and surrounding complex anatomy are a major hurdle that must be accounted for. The more effectively these tools can navigate within this moving environment and the more skilled the surgeon is in their use, the better patient outcomes will be.

Simulation of the surgical environment for such procedures using a physical model has become a popular field of research as it allows the development of new tools, implants, and procedures, or training in the use of existing ones. Further, patient‐specific models have the potential to be used in planning surgical interventions, offering clinicians a tool to determine the best approach for a given procedure, improving patient outcomes. Such models can also be used to better understand cardiac disease, such as valvular heart disease, which presents itself in many different forms, requiring different treatment. Traditional approaches use passive ex vivo animal hearts and heart tissue (such as valve apparatuses) for disease and treatment simulation. These passive specimens are normally attached to mock circulation loops (MCLs) with pulsatile pumps to mimic human circulation. However, in the case of explanted hearts, this external pumping method causes inverse cardiac mechanics, or dilation during systole and contraction during diastole [7, 8], rendering them invalid in most cases. Given the interdependence of the mitral valve and its ventricular anchorage, researchers have also explanted the MV apparatus from animal hearts to perform both surgical and biomechanical simulation [9, 10, 11, 12, 13]. However, by removing the surrounding cardiac structures, they limit themselves purely to simulation of the apparatus itself, neglecting the motion of the beating heart.

Noticing these gaps in ex‐vivo simulation of internal cardiac structures along with the short shelf‐life of ex‐vivo models, Park et al. [14] introduced a groundbreaking approach that preserves both biomechanics and keeps internal structures intact. They stripped away the ventricular musculature of the left ventricle (LV), chemically fixed the internal structures of their porcine model, and replaced the native muscle with artificial, soft robotic muscle fibers (McKibben actuators). The exception was the papillary muscles, which remained passive. A previous in silico study by Park et al. [15] had attempted to find a suitable simplification of human myocardial architecture that would allow their McKibben muscles to generate biomimetic ventricular motion. However, while they were able to reproduce physiological twist and volume magnitudes, their oversimplification to only one helical layer and one circumferential layer, and their actuator choice led to the inability to decouple ventricular torsion from lengthening, and physiologically inverse ventricular lengthening during systole. The same artificial myocardial architecture was used in their hybrid ex vivo/synthetic simulator including their in‐silico study [15]. Further, the peak aortic and mitral flow values reported (∼2 and ∼1 L min^−1^, respectively) are much lower than expected for a porcine heart, indicating the stroke volume (SV) from the simulator may be below physiological expectations. Work by Singh et al. [16] used a similar method to create a soft robotic / ex vivo hybrid model of the right ventricle (RV), where they investigated the addition of a papillary muscle, external to the RV chamber, to induce restriction to tricuspid valve (TV) closure, mimicking restrictive TV pathology. However, since existing chordae/papillary muscles were present, the artificial papillary muscle's elongation could not be used to simulate TV prolapse.

Moreover, all ex vivo methods are fixed to a single set of cardiac geometries, dictated by the animal's cardiac structures, making investigations of patient‐specific or disease‐specific hearts impossible. Researchers have begun to solve this problem using 3D printing and molding technologies to fabricate specific cardiac geometries from patient imaging. Such additive manufacturing methods have been used to fabricate specific static [17] and dynamic mitral valve models [18, 19]. However, these isolated mitral valve simulators carry the same limitations as their ex vivo counterparts because they do not include other cardiac structures and are typically placed inside a rigid box representing the ventricle with a pulse duplicator attached to provide flow. One significant example of patient‐specific, left heart simulation is the work by Rosalia et al. [20], who used 3D printing techniques to fabricate patient‐specific LV and aortic geometries to replicate hemodynamic parameters. However, the actuation of the LV membranes was delivered by inflatable pouches and did not provide biomimetic ventricular motion. Similar to approaches in Park and Singh's work [14, 15, 16], there have been many attempts to recreate the complex motions of the heart by mimicking its muscular architecture [21, 22, 23], but none have included all of the following: fully synthetic, programmable cardiac geometry and internal physiological valve apparatus. It should be noted that a recent preprint by Roche et al. [24] introduced a method of 3D printing patient‐specific models of the LV and left atrium (LA), actuated by the same McKibben muscles in Park and Singh's work. While this work focusses on left atrial function under healthy and diseased contraction patterns, in the lens of our work, its ventricular musculature carries the same limitations described above, and the mitral valve apparatus is simplified to a mechanical valve prosthesis, as with the aortic valve. Omission of the chordae and papillary muscles of the mitral valve apparatus precludes investigations of valvular (dys)function and interactions between ventricular and valve mechanics.

In this paper, we present an in vitro, soft robotic, left heart simulator whose additive fabrication offers the ability to include patient and disease‐specific cardiac geometry. Moreover, its biomimetic, artificial musculature (including two papillary muscles) ensures physiological biomechanics and can encode patient and disease‐specific motion (Figure 1). The simulator's inclusion of molded heart valves, including the mitral valve, its chordae tendinea, and papillary muscles, allows for the simulation and study of valvular disease. We use 3D printed molds of the simplified inner membranes of the human left heart to cast a silicone model, and we place thin‐filament, hydraulic, artificial muscle fibers where they exist in the human heart. This technique allows for the fabrication of patient and disease‐specific, biomimetic, left heart models. In this study, the simplified model replicates key physiological features, including cardiac motion, pressure, and flow patterns. The thin membrane construction also enables compatibility with clinical echocardiographic imaging techniques. Using M‐mode ultrasound imaging, variable mitral valve closure was demonstrated by adjusting the papillary muscle tension, allowing simulation of conditions ranging from proper coaptation to complete prolapse. Color Doppler imaging was also employed to assess mitral and aortic flow, revealing regurgitant jet formation in the prolapsed mitral valve scenario. The impact of mitral prolapse on cardiac hemodynamics was further examined by comparing pressure and flow dynamics to those of normal valve function with pressure‐volume loop features aligning with physiological expectations. The left heart simulator was further used as a testbed for a novel soft robotic catheter equipped with in‐situ contact force sensing capabilities (see Figure S8), allowing performance evaluation in a biomimetic environment. Although the geometry of this model is simplified, this work highlights the potential of fully synthetic and highly controllable biomimetic cardiac simulators for advancing the understanding of heart disease, enabling the development of next generation cardiac devices and providing realistic platforms for in vitro experimentation and training of cardiovascular intervention procedures.

## 2 Results

### 2.1 Design and Fabrication, and Control of the Left Heart Simulator

**FIGURE 2:**
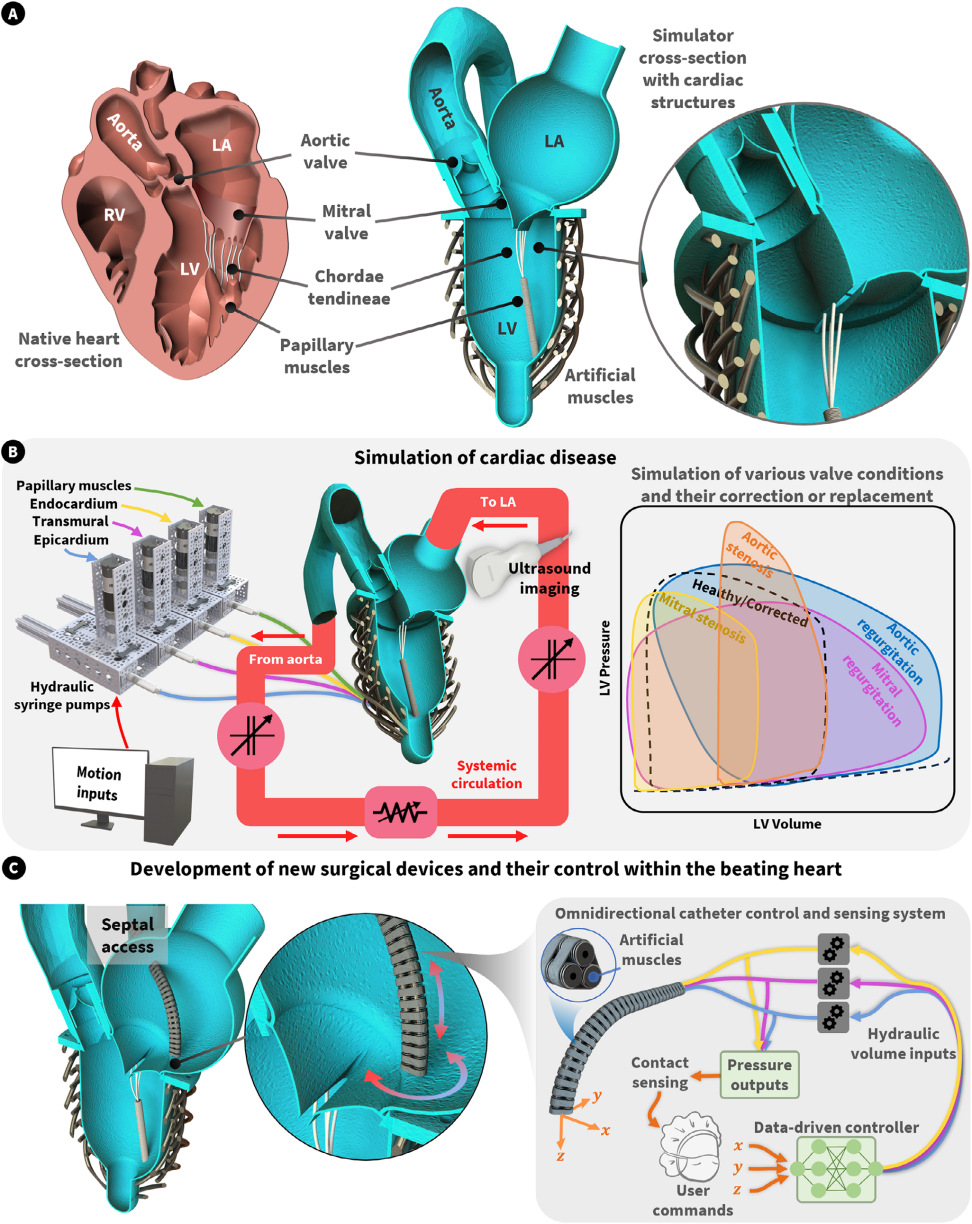
Overview of the design and control of the left heart simulator and its application. (A) bioinspired design of the left heart simulator, complete with internal valving, including the mitral valve apparatus with two papillary muscles (one of which cannot be seen in this cross‐section of the simulator). (B) Control and actuation of the artificial musculature of the left heart simulator and the various potential valve diseases it can simulate. Here is a simple diagram of the MCL used to load the left heart, containing arterial and pulmonary compliance chambers, and a systemic vascular resistance element. (C) The use of the simulator as a platform for the development of new surgical devices and their control within the beating heart. Abbreviations: left ventricle (LV), left atrium (LA), right ventricle (RV).

Figure 2A reveals the geometry and internal structures of the native left heart, including the aortic and mitral valve, with its chordae tendineae and papillary muscles, vital for appropriate function. Here, a simplified model of the native left heart is presented, featuring biomimetic valves and a complete mitral valve apparatus. There are two papillary muscles, one of which cannot be seen in this cross‐section of the device. The details of the fabrication of the inner cardiac membranes (shown in blue here) can be found in the Methods section. It is worth noting that these membranes were cast from silicone using 3D printed molds of simplified cardiac geometries. The left ventricular (LV), left atrial (LA), aortic, aortic valve, and mitral valve membranes were cast individually and glued together using uncured silicone to form the complete left heart inner membrane. In this proof‐of‐concept work, most molds were designed with simplified geometry. Particularly, the aortic mold was designed from a publicly available model, demonstrating the possibility of patient‐specific simulators using our methodology. The artificial musculature was designed and manufactured using a validated method described in our previous work [25]. Figure 2B details the control and actuation of the artificial musculature. We utilized feedforward trajectories to control hydraulic piston pumps that power the hydraulic artificial muscles, which elongate upon pressurization and vice versa. Each layer of the myocardium and the papillary muscles were powered by separate piston pumps. Further, we fit the left atrial input and aortic outlet to a mock circulation loop with variable compliance and resistance elements to mimic the circulation of the human body, which allows us to examine the pressure‐volume relationships of the chambers of the left heart. Leveraging the controllability of the musculature of the simulator (including the two papillary muscles), various disease states can be simulated. Figure 2C shows how the soft left heart simulator can be used as a platform for the development of surgical devices and their control within the beating heart.

### 2.2 General Hemodynamics and Valve Function

**FIGURE 3:**
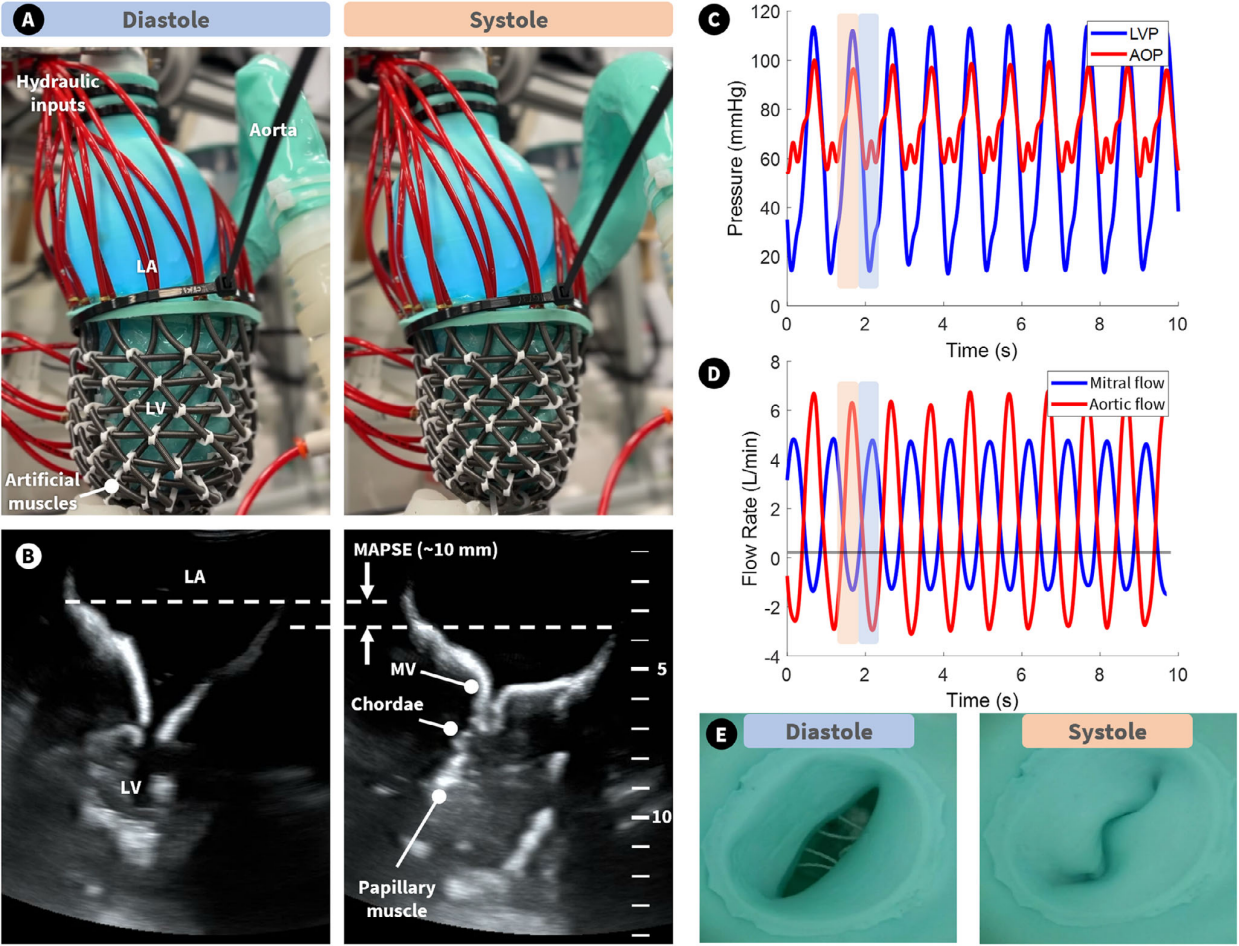
Hemodynamics and MV function in the beating simulator. (A) The left heart simulator was imaged at peak diastole and systole. (B) Ultrasound imaging of the MV apparatus at peak diastole and systole. (C) Left ventricular pressure (LVP) and aortic pressure (AOP) over 10 cardiac cycles. (D) Mitral and aortic flow rate over 10 cardiac cycles. (E) Endoscopic imaging of the MV at peak diastole and systole.

Before investigating the simulation of valve disease, we first evaluated the overall pumping performance of the simulator. Figure 3A shows the external image of the device at peak diastole and systole operating at 60 beats per minute (bpm). There is a clear ventricular shortening and radial contraction, matching with physiological expectations and results from the literature [25]. We then used B‐mode ultrasound imaging to examine the mitral valve apparatus, which is shown in Figure 3B. We observed mitral annular plane systolic excursion (MAPSE) of ∼10 mm, which is in line with clinical expectations, typically ranging from 12–15 mm [26]. It can also be seen that the mitral valve is operating as expected, with clear leaflet separation when the valve opens during diastole, and clear coaptation when the valve closes in systole. We also used an endoscopic camera to further validate this behavior, as shown in Figure 3E. The papillary muscles and chordae tendineae are visible and tensioned in systole. Finally, Figure 3C,D reveals the pressure and flow waveforms for the simulator beating under the same conditions as the imaging. Left ventricular pressure reaches a systolic peak of nearly 110 mmHg which is within the healthy range for humans, while aortic pressure peaks at approximately 100 mmHg with a pulse pressure of around 40 mmHg. The pressure difference between peak ventricular and aortic pressure indicates stenosis or structural stiffening of the aortic valve, which is a common pathology in the human heart. In our model, this condition is simulated thanks to the inherent material stiffness of the artificial aortic valve. This observation demonstrates that material properties of the simulator's valves can be strategically selected to replicate stenotic behavior. While the valve material was not optimally tuned in this work, future work will focus on providing variable valve leaflet stiffness. From Figure 3B,E, it can be seen that the mitral valve does not open to the extent that one might expect from a healthy human mitral valve. This is a further demonstration of relatively high valve stiffness, exhibiting stenotic behavior. Future work is needed to demonstrate that variable valve leaflet stiffness can tune valve function, as considered in the Discussion. The simulator also demonstrates physiological flow waveforms with alternating positive mitral and aortic flow, indicating the proper functioning of the valve structures. A certain percentage of alternating negative flow or regurgitation from both valves is observed, which is to be expected as regurgitation manifests itself even in healthy humans (<30% regurgitant fraction for healthy mitral valves [27]). However, as detailed in the following section, our aim was not to replicate anatomically perfect valves but to develop a method of simulator fabrication that has the potential to replicate patient‐specific geometry and function. The prototype developed here is a proof of that concept, and there is more work to do to demonstrate patient‐specific performance, as outlined in the Discussion. In addition, we demonstrated peak mitral and aortic flow of ∼5 L/min and ∼7 L min^−1^, respectively, more than twice the output of similar existing devices [14].

### 2.3 Effect of Mitral Prolapse on Cardiac Pressures and Flows

**FIGURE 4:**
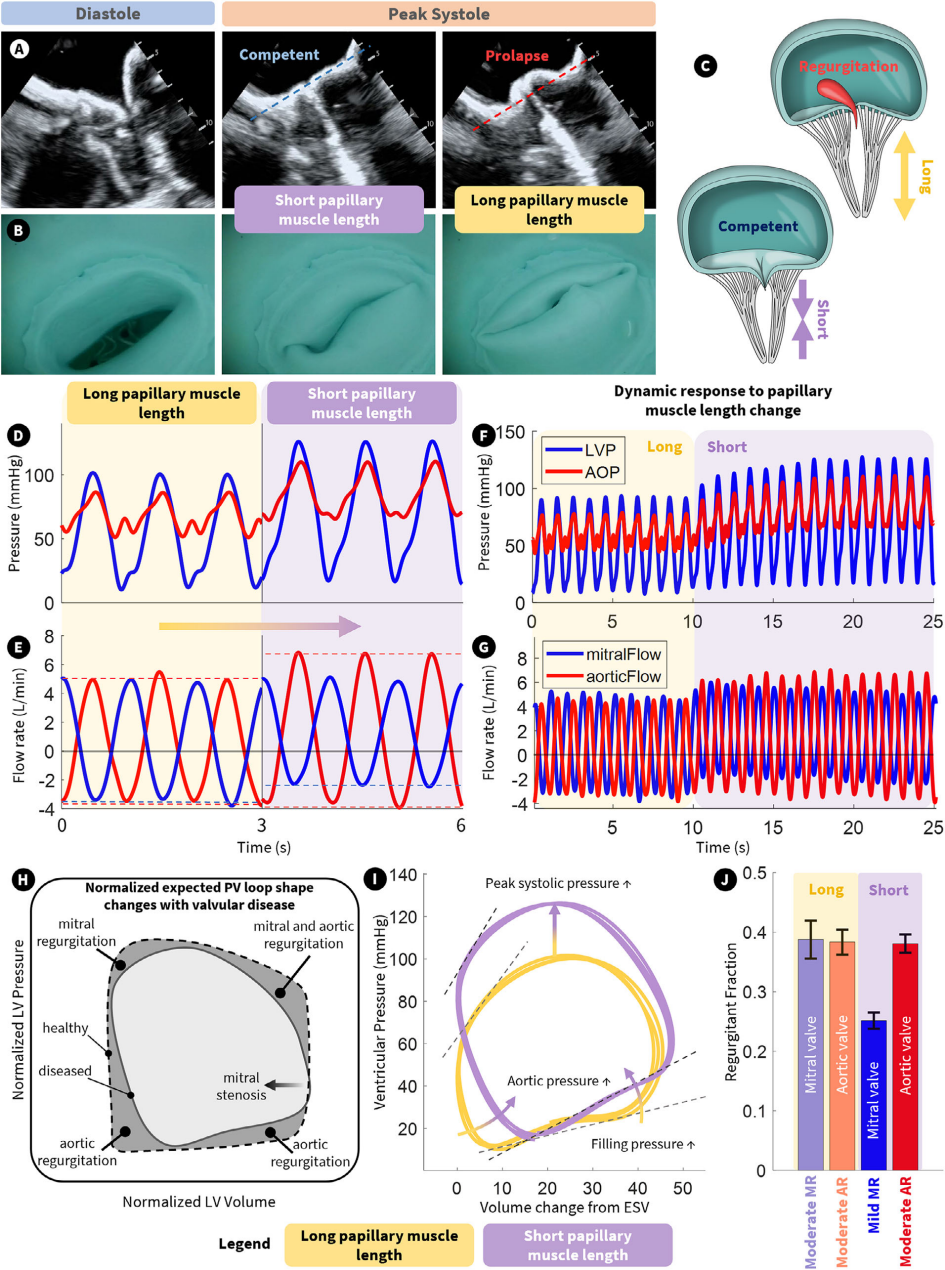
Effect of papillary muscle tension on left heart pressure and flow. (A,B) Ultrasound and endoscopic imaging (respectively) of the MV during diastole and systole with both long and short papillary muscles. (C) Illustration of the effect of changing papillary muscle length on valve competency. (D,E) LVP/AOP and mitral/aortic flow (respectively) with both long and short papillary muscles. (F,G) Dynamic response to the same stimulus. (H) Normalized expected pressure‐volume (PV) loops in healthy and diseased cases. (I) Measured PV loops in the long and short papillary muscle cases. (J) Regurgitant fraction from aortic and mitral valves in the long and short papillary muscle cases.

After validating the overall biomechanics and hemodynamics of the simulator, we leveraged the controllable artificial papillary muscles to induce mitral valve dysfunction and assess its impact on intracardiac pressure and flow of the simulator. Mitral regurgitation occurs when the blood leaks backward into the left atrium due to improper closure of the mitral valve. This disease affects at least 24 million people worldwide [28] and is typically caused by degenerative changes to the mitral valve apparatus. This can include chordal elongation/rupture, annular/ventricular dilation, papillary muscle rupture/elongation, or changes to leaflet geometry, which is generally characterized by resultant backflow across the valve from the LV to the LA, driven by the pressure gradient during systole. Our simulator's ability to adjust papillary muscle length enables the replication of various degenerative changes, specifically those that alter the distance between the origin of the papillary muscle and the insertion of the chordae on the valve leaflets during systole. As shown in Figure 4, changing this length allows the mitral valve leaflets to prolapse into the LA, preventing proper coaptation, causing regurgitation.

In this experiment, we kept the left heart beating within the mock circulation loop, while altering the length of the artificial papillary muscles and monitoring the pressure in the left ventricle (LVP) and aorta (AOP), as well as the flow in and out of the heart (mitral and aortic flow). Adjustments to the papillary muscle lengths throughout this study were done using human‐in‐the‐loop control, through operator inputs with ultrasound/echocardiographic imaging as the feedback parameter. In the lens of the future, patient‐specific, left heart models, whose valve leaflet geometry, cardiac motion, and hemodynamics will differ across samples, we envision that this human‐in‐the‐loop control will enable replication of patient‐specific valve function. An operator can compare in‐vitro echocardiographic valve imaging with imaging taken from patients in‐vivo and adjust arbitrary papillary muscle length to achieve the best approximation of patient valve function. This control loop is illustrated in Figure S11.

**FIGURE 5:**
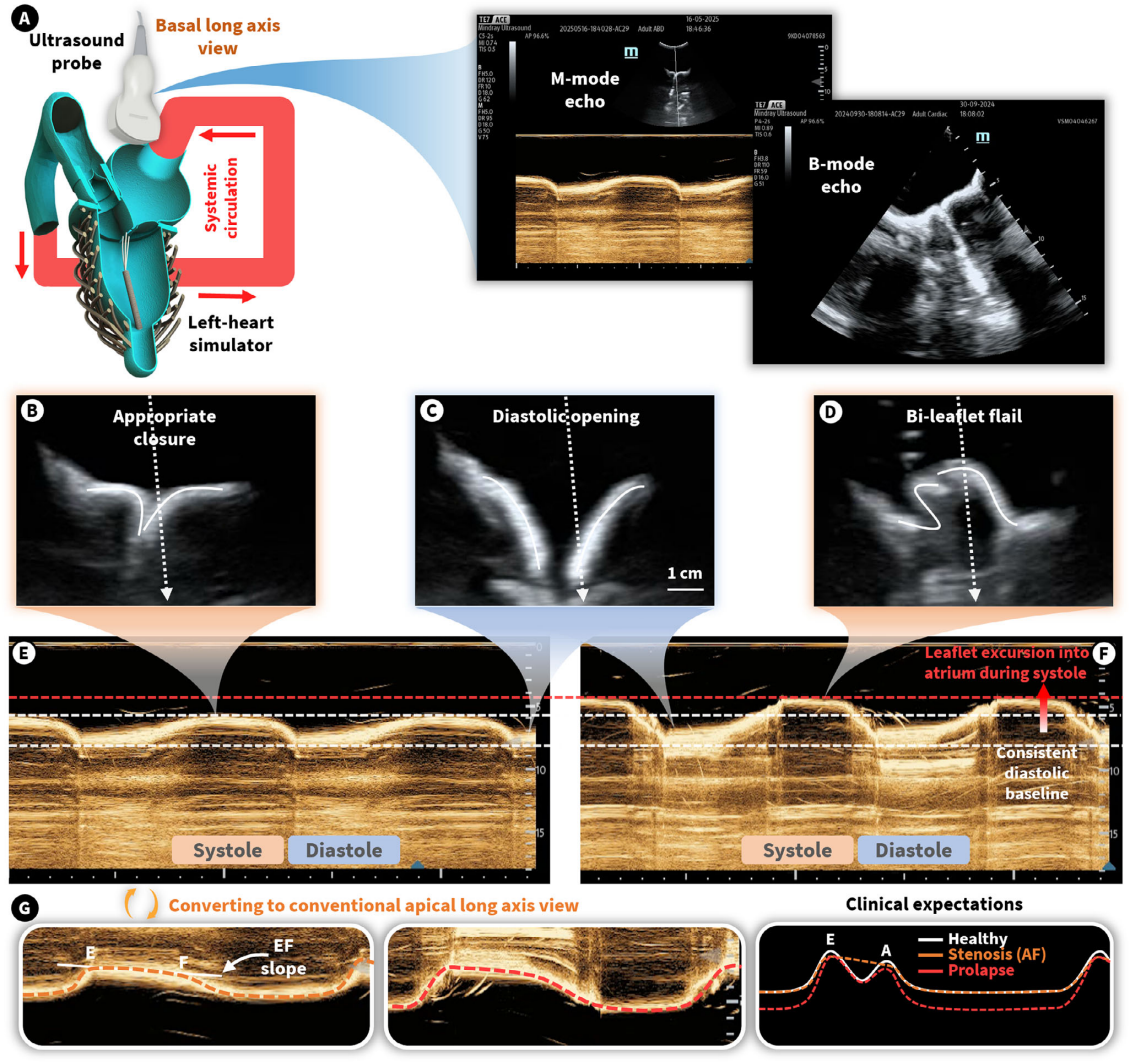
M‐mode ultrasound imaging of the MV. (A) Experimental setup for clinical imaging of the simulated MV. (B–D) B‐mode imaging of the MV leaflets with appropriate closure, diastolic opening, and bi‐leaflet flail, respectively. (E,F) M‐mode imaging of the anterior valve leaflet with appropriate closure and bi‐leaflet flail, respectively, from a basal long‐axis view. (G) The same M‐mode imaging flipped to convert it to the conventional apical long axis view, along with clinical expectations, where EF slope refers to the early diastolic closing velocity of the anterior leaflet before the A‐wave.

Figure 4A,B shows ultrasound and endoscopic vision of the mitral valve during diastole and peak systole with both short and long papillary muscle lengths, while Figure 4C illustrates the effect of changed papillary muscle length on regurgitation. This echo imaging shows a clear separation of the valve leaflets as it opens during diastole, as physiologically expected. When the papillary muscles are kept sufficiently short, the mitral valve has good coaptation, maintaining competency during systole. When the papillary muscle is lengthened from this position, the valve clearly prolapses during systole and travels into the LA, causing regurgitation. While more prolapse may be expected, this papillary muscle length is not the limit of its range of motion, and Figure 5D below shows that it can be further increased to produce leaflet flail. Figure 4D,E reveals the pressure and flow effects of this valvular dysfunction. In the long papillary muscle case, peak LVP only reaches ∼100 mmHg. This is due to the mitral valve regurgitating during systole, and it is unable to hold the pressure generated by the LV musculature. This regurgitation can be seen in the negative mitral flow during systole and the regurgitant fraction shown in Figure 4J, which is ∼0.4 for the mitral valve (placing it in the moderate‐to‐severe category). There is also a clear difference in the peak LVP and AOP for all cases, which indicates aortic valve stenosis. As previously noted, this effect arises from the material properties of the cast silicone aortic valve. Using softer or more compliant materials could help reduce valve stiffness and better mimic physiological behavior. When the papillary muscle is shortened to make the mitral valve more competent, peak LVP is increased to healthy levels ∼120 mmHg and aortic flow increases since the regurgitant fraction (Figure 4J) or backflow during systole (Figure 4E) has decreased. mitral valve regurgitant fraction dropped from moderate‐to‐severe to mild (∼0.25). It is worth noting that the modelled aortic valve exhibits moderate regurgitation in both cases (Figure 4J). In the presence of a competent mitral valve, the increase in aortic pressure increases aortic regurgitation with increased ventricular filling pressure during diastole.

This phenomenon is clearly seen in the pressure‐volume (PV) loops in Figure 4I, which is plotted next to normalized clinical expectations for valvular disease in Figure 4H. There is a clear upward shift and an increase in the gradient of the filling line of the PV loop when the papillary muscles are shortened, indicating increased filling pressures from aortic regurgitation. There is also clear rounding of the corners of the PV loop in keeping with expectations of mitral and aortic valve regurgitation. It should be noted that the left ventricular musculature was driven with consistent, feedforward control for both the long and short papillary muscle cases. This indicates that the ventricular volume in the PV loops remains consistent, although the increased filling pressure slightly raises the end‐diastolic volume in the case of a competent mitral valve. In the human heart, valvular incompetence alters preload and afterload, typically leading to changes in the end systolic and end‐diastolic volumes as the myocardium stretches in response to increased pressure. Future work will focus on implementing closed‐loop control of the artificial myocardium to dynamically adjust ventricular volume in response to internal pressure changes. Supporting Movie S1 shows the effect of continuously increasing and decreasing papillary muscle length on mitral valve leaflet motion using B‐mode echo recording.

### 2.4 M‐Mode Mitral Valve Imaging With Induced Dysfunction

After establishing the hemodynamic characteristics of both healthy and diseased mitral valve mechanics, we employed clinical imaging to assess the valve's motion under each condition. M‐mode ultrasound imaging is commonly used in clinical practice to assess the motion of cardiac structures over time. Figure 5A shows the experimental setup for this imaging method, where an ultrasound probe (Mindray TE7 Ultrasound System) is placed on the left atrium to provide a basal, long‐axis view. This view is equivalent to that seen with transesophageal echocardiography, which is the preferred imaging platform while performing mitral valve procedures, as shown in Figure 5E–G.

While the LV was actively beating within the mock loop, we used M‐mode ultrasound imaging to track the motion of the anterior leaflet of the artificial mitral valve through systole and diastole. During this process, we adjusted the length of the artificial papillary muscles to produce both appropriate systolic closure and complete prolapse, or bi‐leaflet flail. Figure 5B,D shows the B‐mode imaging of the mitral valve leaflets at peak systole with both proper coaptation and complete prolapse, respectively. Figure 5C shows the diastolic state shared by both cases, and Figure 5E,D reveals the M‐mode data for both cases, with clear upward motion of the leaflets during systole and downward motion during diastole. There is a leaflet excursion into the LA in the case where the papillary muscles are lengthened, indicating prolapse. However, the diastolic leaflet position remains consistent in both conditions. Figure 5G presents the basal long axis view flipped to the commonly used apical long axis view allowing easier comparison of leaflet motion waveforms with clinical expectations. In this view, the systolic atrial excursion of the leaflet in the prolapse case is clearly visible. In both cases, the EF slope of leaflet motion is approximately 17 mm s^−1^ and the mitral leaflet separation index is less than 0.5 cm, indicating severe stenosis [29]; where EF slope refers to the early diastolic closing velocity of the anterior leaflet before the A‐wave. This can be explained by the material stiffness of the silicone used to cast the leaflets, which can be adjusted for desired leaflet stiffness. The increased resistance to LV filling caused by this mitral stenosis further justifies the relatively small stroke volume produced by the ventricle (Figure 4I). We also note that this model does not include atrial musculature, meaning that the A‐wave leaflet motion is not currently simulated. This explains why it cannot be seen when compared to clinical expectations in the healthy case (Figure 5G). Our simulator more closely mimics the atrial mechanics of atrial fibrillation (AF) with no global atrial contraction. Movie S2 visualizes the M‐mode imaging as the simulator beats for both competency cases.

### 2.5 Color Doppler Mitral and Aortic Valve Imaging

**FIGURE 6:**
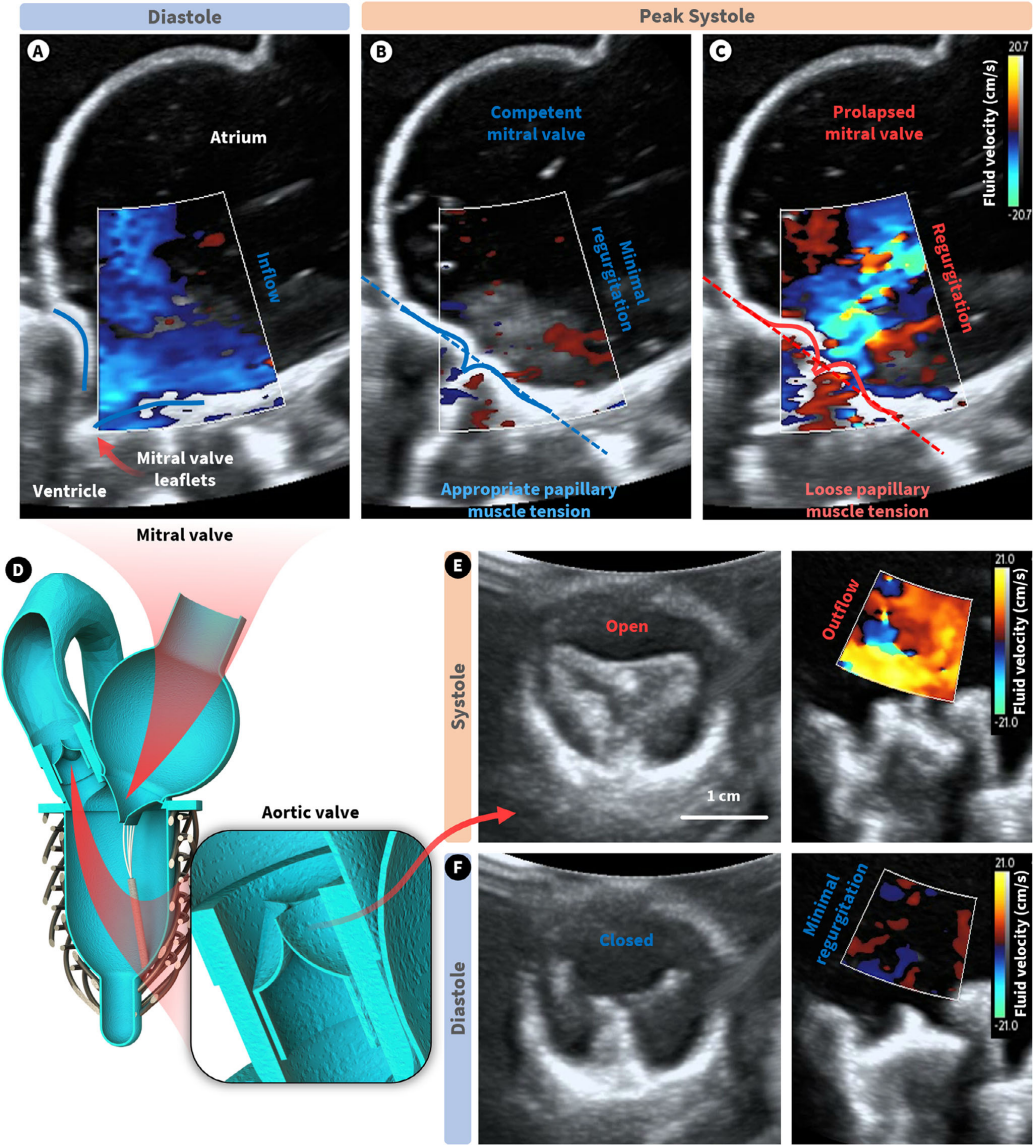
Color Doppler imaging of the left heart simulator. (A) Diastolic flow through the MV from LA to LV. (B,C) Peak systolic mitral flow for a competent and prolapsed MV, respectively. (D) Cross‐sectional illustration of the left heart simulator and the location of its valves. (E,F) The aortic valve in B‐mode radial cross‐section, and color Doppler longitudinal cross‐section in systole and diastole, respectively.

Another common clinical diagnostic tool for cardiac disease is 2D color Doppler ultrasound imaging. This allows clinicians to examine the blood flow through the heart and its structures relative to the ultrasound probe. Here, we investigated the presence of regurgitation in both the competent mitral valve and prolapsed condition, controlled by adjusting the length of our artificial papillary muscles. Figure 6A shows mitral inflow through the valve leaflets during diastole, which appears consistent across both valve competency conditions. In both cases, peak particle velocity at the annulus reached approximately 10 cm/s. During systole, the competent valve with appropriate papillary muscle length exhibited minimal regurgitation upon valve closure (Figure 6B). However, in the prolapsed case, characterized by elongated papillary muscle length, a distinct regurgitant jet was observed flowing back through the prolapsed leaflets (Figure 6C). We also looked at the function of the aortic valve in systole and diastole through the lens of color Doppler imaging (Figure 6E,F). Clear turbulent outflow was observed during systole, with relatively little backflow during diastole. However, the stenotic behavior of the aortic valve is further demonstrated by its limited opening area (<1 cm^2^), consistent with the hemodynamic assessment in previous sections. Movies S3 and S4 show recordings of the flow through the aortic and mitral valves, respectively.

### 2.6 The Left Heart Simulator as a Testing Platform for New Soft Robotic Catheter

**FIGURE 7:**
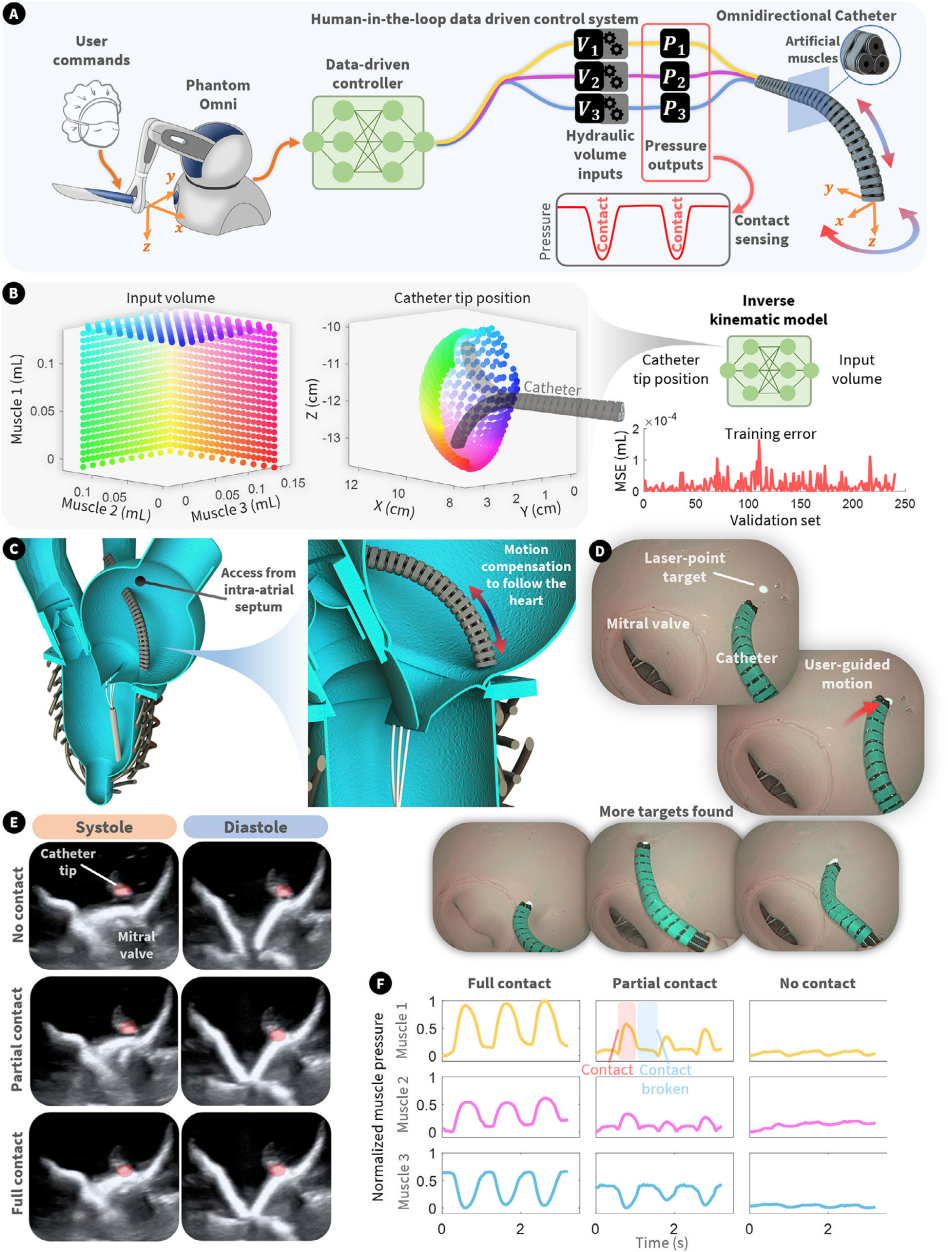
The left heart simulator as a platform for the development of surgical tools. (A) Overview of the control of the soft robotic catheter. (B) Input (hydraulic volume) and output (catheter tip position) data used to train the inverse kinematic model, along with the training error. (C) Depiction of the insertion of the catheter into the simulator and its positioning in the left atrium. (D) Endoscopic vision of the user‐controlled catheter operating within the simulator and moving to various point targets imposed by a laser pointer from outside the simulator. (E) Echocardiographic imaging of the catheter within the beating simulator through systole and diastole in three contact states (none, partial, and full). (F) Muscle pressure readings from the catheter in three contact states (none, partial, and full).

Finally, we demonstrated the use of the simulator as a benchtop platform for the development of a soft robotic cardiac catheter with self‐force sensing capabilities. The structure and working principle of the catheter are illustrated in Figure S8 and detailed in Methods. Traditional cardiac catheters are typically pre‐curved or tendon‐driven, requiring significant training for effective use. There is growing interest in leveraging data‐driven models to simplify control and improve the usability of cardiac catheters. Here, we have developed a simple inverse kinematic model of a hydraulic artificial muscle‐driven soft robotic catheter, capable of omnidirectional motion (Figure 7A). The model details are outlined Methods. This model takes a desired catheter tip position in 3D space and predicts the correct hydraulic input volume for each of the catheter's three artificial muscles to achieve that position (Figure 7B). To enhance the intuitive control of the catheter, we employed a Touch Haptic Device (3D Systems) to capture the user's desired tip position and relay it to the inverse kinematic model. To constrain the Touch's cursor within the workspace of the catheter, we also used a haptic force field, equipping the user with a haptic boundary and a better feel for the manipulation of the catheter. Although the catheter is capable of omnidirectional movement, we constrained it to pure bending for the demonstration. It is worth noting that incorporating elongation introduces redundancies into the data‐driven model that are nontrivial to resolve. This challenge will be addressed in our future work. In this demonstration, the elongation of the catheter was controlled by the user's insertion and retraction of the surgical tool, consistent with standard practice for conventional cardiac catheters.

Figure 7C shows the insertion of the catheter into the simulator via the intra‐atrial septum wall and its positioning within the left atrium. This is a common access point for endovascular procedures of the left heart, including TEER and cardiac ablation. An endoscope was also inserted in the atrium to analyze the motion of the catheter. Figure 7D shows images captured by the endoscope. We used a laser pointer to impose point targets onto the atrial wall visible to the endoscope and the user. As depicted, the user attempted to move the catheter's tip to the target using the Touch Haptic Device in various positions. Videos of these target operations can be found in Movie S5. This demonstrates the utility of the simulator in the evaluation of tool performance. While not the focus of this paper, such simulators can provide valuable metrics for the iterative development of new surgical tools and their control, such as assessment of cognitive load improvements with more intuitive control. Similarly, they can also be used to train and evaluate a surgeon's performance when using existing surgical tools.

Further, the soft robotic catheter powered by fluid‐driven artificial muscles has the inherent ability to sense environmental changes by monitoring internal fluid pressure of the soft artificial muscles while the user's input remains constant. Within both the real heart and our simulator, these environmental changes may arise from variations in fluid flow, pressure, and contact with cardiac structures. Depending on the specific interventional task, such as ablation, electrical mapping, or implant delivery, this sensory feedback can be leveraged to enhance the procedural accuracy and clinical outcomes. Figure 7F demonstrates the catheter's ability to sense various contact signals, where the pressure readings from the catheter's constituent artificial muscle fibers are shown under three different contact conditions (see Figure 7E). Ultrasound imaging shows three different contact conditions between the catheter and the mitral annulus. In the ‘no contact’ case, the catheter remains untouched as the mitral annulus descends during systole and ascends during diastole, respectively. ‘Partial contact’ refers to separation during systole with contact occurring as the annulus rises during diastole. ‘Full contact’ is defined by continuous contact throughout both systolic descent and diastolic ascent, maintained by the catheter's soft body deformation. The pressure reading in Figure 7F shows minor oscillations under the “no contact” condition, likely resulting from dynamic pressure and flow changes within the left atrium during the simulator's beating cycles. In the “partial contact” case, distinct pressure peaks occur when the catheter briefly contacts the heart, followed by a distinct flat spot when contact is lost. In contrast, “full contact” produces an amplified near‐sinusoidal wave pattern, maintained by continuous interaction with the cardiac structure. Movie S6 further demonstrates the contact sensing capabilities. Notably, the difference between full and partial contact suggests that the pressure signal's duty cycle could be used to infer the degree and consistency of contact between the catheter and the heart.

## 3 Discussion

This paper presents a fully synthetic, biomimetic soft robotic left heart simulator, featuring a functional and tunable mitral valve apparatus. Using 3D printed molds, the geometry of its artificial cardiac membranes can be designed for patient/disease‐specific cases. Notably, the cast soft material used in the simulator can be tailored to achieve the desired stiffness of cardiac structures such as valve leaflets. Its compatibility with clinical imaging modalities, including echocardiographic imaging, enables detailed investigations of the biomechanics and hemodynamics of valvular disease. Further, the simulator's biomimetic structures and motion offer a stable and controllable platform for the evaluation and development of new surgical tools and their control systems, as well as training for existing cardiac devices. In this study, we demonstrated the simulator's ability to replicate physiological ventricular pumping, characterized by ventricular shortening and radial constriction during systole. This biomimetic contraction supports proper internal valve function, with realistic MAPSE values and mitral/aortic valve leaflet dynamics, resulting in pressure and flow waveforms that closely mimic those observed in the native heart.

Having demonstrated our simulator's ability to reproduce left heart function, we employed the variable length control of the two papillary muscles in the mitral valve apparatus to modulate mitral valve competency. Once elongated sufficiently to achieve proper leaflet coaptation, further elongation of the papillary muscles induces prolapse into the LA. We used B‐mode, M‐mode, and color Doppler echo imaging, all standard clinical diagnosis techniques, to assess the performance of the mitral valve. The proof‐of‐concept cardiac membrane was fabricated using Smooth‐Sil 960 (Smooth‐On) silicone. Due to the selected material and casting thickness, the resulting aortic and mitral valve leaflets were stiffer than those found in a healthy human heart. While this material choice can be optimized to model healthy leaflets in the future, it unintentionally produced clinically relevant manifestations of both aortic and mitral valve stenosis, which we successfully diagnosed using our simulator and clinical criteria. Aortic stenosis was identified via the LV/aortic pressure gradient and a restricted aortic valve opening area observed in B‐mode echo. Mitral valve stenosis was diagnosed using B and M‐mode echo by analyzing pathological leaflet motion and diastolic separation. In addition to these material‐induced pathologies, we successfully induced and identified mitral valve prolapse through controlled elongation of the papillary muscle length. Systolic excursion of the mitral valve leaflets into the LA was detected using B and M‐mode echo imaging, while color Doppler echo imaging visualized the resulting systolic regurgitant jet. Future work will focus on refining the material selection during the fabrication process to improve the physiological fidelity and versatility of the simulator. This could include varying the stiffness/thickness of leaflet material to induce healthy and diseased states. This material optimization could also be extended to other regions of the simulator, such as the aorta and the aortic root, to simulate and examine their biomechanics/hemodynamics in both health and disease. Further, this platform could be used to assess the performance of new artificial heart valves and their minimally invasive implantation. Beyond material selection and leaflet thickness/geometry, it would also be interesting to explore active methods of valve leaflet stiffness modulation. Similar to the human‐in‐the‐loop control over papillary muscle function, if active leaflet stiffness changes could be applied, a user could adjust parameters to change valve function in real time using echo imaging as feedback. In this way, the user could change valve stiffness to best match patient‐specific valve function from their echocardiographic imaging. Various soft robotic variable stiffness mechanisms could be employed to achieve this (electrothermal, fiber‐jamming, fluidic actuation, etc.), although thin‐film fabrication presents challenges.

While this work is an important first step towards robust, patient/disease‐specific, in vitro modeling of the beating heart and its structures, improvements can and should be made. One of the limitations of this work is the omission of the atrial musculature. The addition of this contraction would improve the biomechanics of the simulated mitral valve, which currently lacks A‐wave motion. This A‐wave flow (from atrial contraction) also improves the pumping efficiency of the heart, increasing diastolic filling, which could help to increase stroke volume in our simulator. Notwithstanding this limitation, it should be acknowledged that many patients with mitral valve disease have atrial fibrillation, without any atrial mechanical contribution to left ventricular filling. Further, the placement of the origin of our simulated papillary muscles was perhaps more apical than the average human heart. This was done for design simplicity, as our hydraulic input to the muscles could then fit into the ventricular myocardium more easily. However, this papillary muscle placement limits the range of mitral valve dysfunction that can be simulated. While lengthening of the artificial papillary muscles could simulate the effects of chordal elongation/rupture and papillary muscle rupture/elongation, the effects of ventricular dilation could not be simulated properly. For example, eccentric and concentric remodeling can change the position of the origin of the papillary muscles relative to the mitral valve annulus, and so can ischemic tissue death. Through the chordal tether, this can pull the mitral valve leaflets in a range of directions, each producing a different mitral valve dysfunction. Moving the origin of our artificial papillary muscles from the apex and closer to the base of the ventricle would allow simulation of radial displacement caused by such ventricular disease. Such improvements would enhance the anatomical/physiological accuracy and utility of our simulator, taking advantage of the control we have over ventricular mechanics with our unique myocardial architecture.

While we don't believe it detracts from the findings of this study, it should be reiterated that the LV and LA geometry used here is idealized (the LA is also oversized). Future work must include replication of patient‐specific geometry, and the performance of these models must be validated against patient data. The work presented here provides a fabrication methodology that makes this possible. The envisioned future workflow from patient imaging to model fabrication can be seen in Figure S10. Further, some silicone‐cast components were too thick for ultrasound penetration. As a result, it was easier to image the LA and mitral valve, but harder to image the LV cavity. Future iterations will include thinner membranes or different material selection for better echo imaging. We have also included echo imaging of a new membrane design iteration, which can be better penetrated in Figure S5. The silicone used in this study was not doped for ultrasound image quality, it was imaged without modification.

Given the rising demand for minimally invasive cardiac interventions, we present our simulator as a stable and controllable platform or environment for the development of new surgical tools and their control systems. It is important to note, however, that further work is needed to develop cardiac membranes that exhibit similar frictional properties to that of the native myocardium. The current silicone membranes were not optimized for this purpose, and it is likely that interactions between the novel catheter we tested and the inner surface of the simulator do not accurately represent those of the native heart. While we presented early validation of novel catheter performance (navigation and simple contact sensing) more mature devices need more realistic surface properties to assess detailed control and performance parameters. This not only includes contact dynamics of end‐effectors, but also of device anchoring in the heart, none of which were investigated in this study. Future work in this area could include silicone surface treatment or coating. Micro‐texturing of molds, for example, could modulate the frictional properties of the cast membranes, or methods of adhering hydrogels to the membranes could be developed and implemented. Alternatively, hydrogel materials could be considered for the casting process, rather than silicone. Addressing this limitation will enable our simulator, and others that follow, to produce a simulation environment approaching that of the native heart.

Further, for the future development of simulators such as this, it is crucial to consider closed‐loop control algorithms to regulate preload/afterload sensitivity. While the feedforward control approach used in this study is somewhat mitigated by the natural preload/afterload sensitivity exhibited through the soft robotic actuators (characterized in our prior work [25]), closed‐loop control is needed for true control over cardiac (dys)function. Neglecting this means the simulator does not respond to hemodynamic loading in physiologically appropriate or controllable ways. Integration could be as simple as real‐time chamber pressure residual PID, but more sophisticated methods could involve sensorization of the myocardium itself to control stress and strain. We also investigated the artificial muscle's force‐length and force‐velocity relationships, and loaded frequency response, with respect to mammalian myocytes, which can be seen in the Supporting Information. The purpose of this investigation was to compare out artificial muscles to real ones and comment on our artificial muscle's suitability for cardiac simulation as a result. It revealed that our artificial muscles are faster and stronger than mammalian myocytes, when normalizing for fiber cross‐sectional area and length. This ability to contract faster than myocytes at higher loads invites the opportunity to implement closed‐loop control algorithms that scale down these speeds and applied forces to match the specific behavior of natural myocytes. Extrapolating from the individual muscle fibers to the artificial myocardium, this means recapitulation of native biomechanics is indeed possible, and it is something we are actively pursuing.

Along with closed‐loop control, it is important to recognize that the current myocardium responds to input commands by contracting or relaxing each of the three muscle layers in its entirety. While this is sufficient to model global ventricular motion, specific disease modeling may require recapitulation of segmental wall motion. This would be particularly valuable for the simulation of, for example, mitral valve function after myocardial infarction, since its leaflets may be tethered to a section of dead myocardial tissue, which behaves differently to healthy myocardium. Hearts can also remodel in unique ways following insult, meaning segmental control over the artificial myocardium could potentially enable simulation of remodeling processes and their effects on the left heart's function. While closed‐loop control over entire layers of the artificial myocardium can facilitate simulation of the global effects of diastolic and systolic dysfunction, segmental control could offer a far greater range of high‐fidelity simulation. That said, segmenting muscle fibers within the myocardium to the required resolution calls for more advanced, automated fabrication, rather than hands‐on methods. Multi‐material printing, as we discuss below, could hold the answers by enabling hard/soft 3D structure generation of individual, segmented muscle fibers at programmable fiber angles, and could also allow the integration of hydraulic input routing throughout the structure. Further, along with improved fabrication, the current actuation involves individual hydraulic syringe pumps for each muscle group, which becomes impractical when scaling the number of individually controlled fibers within each group. As with many pneumatic systems, we would need to pivot to the implementation of a hydraulic pressure source that is distributed to each muscle fiber/group via controllable valves.

Finally, the ultimate goal of this work is a path to translation and clinical acceptance to improve the delivery of cardiovascular healthcare. There are several specific limitations of this work that need to be addressed for this to eventuate. Firstly, improvements to the fabrication processes are needed to improve both speed and the elimination of human error. The cardiac membranes described in this work are perhaps the easiest to improve, and we are confident that advances in 3D printing technologies and materials will provide excellent alternatives to the casting processes we described. While we have experienced issues with the tear resistance of some currently available materials, such as elastic SLA resins, there is a great deal of R&D being focused on alternative printing methods and materials. With accurate printing, highly elastic materials, and multi‐material methods, cardiac membranes with tunable mechanical properties can be imagined. Further, the fabrication of the artificial myocardium and its muscles is labor‐intensive, with great potential for human error. While we are constantly improving the process to reduce hand‐on steps, we are excited about advancements in hard/soft multi‐material 3D printing technology. Future developments could enable automated production of both the elastomeric components of the musculature and the stiff constraints that allow it to move along desired muscle fiber directions.

These manufacturing and material choices must also be considered with clinical imaging compatibility in mind. We believe that hydraulic actuation is a good choice regarding echocardiographic imaging, for example, since pneumatics can block echo signals, leading to image distortions. However, the metallic coils currently used in our artificial muscles must be replaced with polymeric constraints to reduce unwanted image artefacts and compatibility with X‐ray and magnetic resonance imaging. Multi‐material polymeric fabrication, as described above, is a promising direction to action this. We do however believe that the form factor of the simulator is appropriate for most imaging techniques (assuming appropriate material choice). It can be placed in a water bath to allow ultrasound probe viewpoints that simulate those used in practice. Similarly, it can be oriented within the view of a C‐arm CT machine, for example, to reproduce images typically used for device navigation.

Looking to the future, building trust in this platform, and others like it, as a predictive tool for surgical outcomes, is the next necessary step towards clinical adoption. This means rigorous validation against in‐vivo data. Beginning with retrospective datasets from humans, pre and post operative imaging and hemodynamic data need to be reproduced in‐vitro, with robust success across many patients. This would demonstrate an ability to predict procedural outcomes. From there, it would be valuable to demonstrate real‐time use of such models to prospectively guide interventions. Beginning with animal models, success here in predicting outcomes would point the way towards clinical adoption.

This simulator and its future iterations provide biomimetic form and function of the inner surfaces of the left heart, and its 3D model‐guided fabrication has the potential to include patient/disease specific geometries. Further, our artificial myocardium can be tuned to move and pump fluid as desired. We recognize that the inner membranes in this work were modelled on simplified cardiac geometries, but this can be adjusted in future work and doesn't take away from the new findings of this paper. Additionally, we recognize the importance of accounting for superimposed cardiac motion caused by respiratory cycling, which was not accounted for in this study. That being said, our simulator, consisting of soft robotic muscles, can potentially simulate the effect of extracardiac forces, such as those from the lungs and diaphragm, which will be included in future work. More work is also needed to scale down the control and actuation unit, along with the mock circulation loop, as discussed in the Methods section. Scaling these components will allow for portability and potential use in clinical environments.

In this work, we also demonstrated the assessment of an omnidirectional, soft robotic, cardiac catheter with contact sensing capabilities, and a data driven controller for intuitive and potentially automated interfacing. We demonstrated the catheter's ability to move within the LA of the beating simulator and novice users were able to reach targets successfully. Further, we were able to demonstrate that the catheter could sense contact with the mitral valve annulus as it moved up and down through the cardiac cycle. In addition, we observed the catheter's ability to distinguish between different types of contact (partial and full) by analyzing the duty cycle of the resultant pressure waveforms from its hydraulic channels. This type of high‐fidelity testing is invaluable during the early stages of device development, not just in the design, but in control and sensing, to deliver better user experience and outcomes. Future research will investigate the use of simulators like this to train surgical robots to autonomously operate within the beating heart.

## 4 Conclusion

In summary, this paper introduces a new method for fabricating a fully synthetic, biomimetic left heart simulator. The simulator's inner membranes and cardiac structures (including valves) are cast using 3D printed molds, allowing the geometry to be programmed to match patient/disease specific forms. The artificial myocardium is powered by thin filament, hydraulically driven artificial muscles arranged in a biomimetic, three‐layered architecture, enabling replication of the complex motions of the cardiac cycle. The simulator also features controllable artificial papillary muscles capable of inducing both competent and diseased mitral valve function. Its compatibility with echocardiographic imaging allows for the application of clinical diagnostic criteria to assess cardiac performance and disease states. As such, this platform can be used to investigate the hemodynamics and biomechanics of various cardiac pathologies. Further, under simulated diseased conditions, this model offers a robust and controllable platform for the development of new surgical devices and control strategies to treat those diseases. Fully synthetic, biomimetic cardiac simulators like this hold significant promise for enhancing procedural training, optimizing cardiac device design, and ultimately improving clinical outcomes through more intuitive and potentially automated control of interventional cardiac devices.

## 5 Methods

### 5.1 Design and Fabrication of the Simulator

The ventricular musculature was designed and fabricated using the same methodology as in our previous work [25]. However, to improve the actuation speed and reach 60 bpm, we segmented each layer of muscle, increasing the number of hydraulic inputs into each layer. We increased the number of inputs into both the endocardial and epicardial layers from 2 to 12. Similarly, we increased the number of inputs into the transmural layer from 2 to 7. In doing so, we reduced the resistance to input fluid into the muscles, allowing for faster actuation of the musculature. The papillary muscles were constructed in a similar way to those in the artificial myocardium, instead of using metallic springs to constrain their inner rubber tubes, we used wrinkled fabric sheaths. They function in the same way; however, the fabric of the papillary muscle sheaths will not rust in the fluid‐filled ventricular chamber, while the steel coils used in the ventricular musculature will rust with prolonged exposure to water. The silicone membranes of the simulator were all cast from Smooth‐Sil 960 (Smooth‐On) and were all fixed together using uncured Smooth‐Sil 960 as a glue. Each component of the inner membrane assembly was cast using 3D printed molds, which were modeled from simplified cardiac geometry. The aortic mold, however, was designed from a publicly available model (Grab CAD) of a human aortic arch, as a demonstration of the integration of patient‐specific cardiac structures. The details of the assembly and molding of the inner cardiac membrane can be seen in Figure S7. The mitral and aortic valve leaflets were fabricated through dip coating 3D printed molds; they were dipped 2 times, reaching a leaflet thickness of ∼0.7 mm. The leaflet thickness and material properties were not optimized for performance metrics and should be improved in the future. Similarly, the LV and LA membranes were cast ∼1 mm thick. Before gluing components together, the papillary muscles were attached to the MV leaflets via chordae tendineae (yarn) using Smooth‐Sil 960 as a glue. The proximal ends of those muscles, with their hydraulic input lines, were threaded through holes in the LV membrane located near its apex. Then the assembly could be fixed together, and the joint between the papillary muscles and the LV membrane was fixed using Smooth‐Sil 960 as a glue. The LV musculature could then be slid over the LV membrane, threading the papillary muscle fluid transmission lines through the apex of the LV musculature, and fixed in place with Smooth‐Sil 960. While the prototype used in the experimentation of this paper utilized a two‐part mold to create a thick cardiac skeleton Figure S7E, this thickness causes undesirable ultrasonic shadowing. In Figure S7F, we suggest a method of creating a thin cardiac skeleton with an integrated MV. We created a prototype of this membrane to demonstrate much improved ultrasonic penetration and therefore imaging (Figure S5).

### 5.2 Control and Actuation of the Simulator

While the myocardium was segmented into multiple fluid transmission inputs compared to previous work, each of the three layers of the myocardium was still powered by one piston pump, whose output was split using 3D printed manifolds (Form 4 SLA printer, Formlabs). The piston pumps were custom‐built using DC motors and fittings from goBILDA (USA) and controlled by RoboClaw motor controllers (Basicmicro Motion Control, USA). These pumps were designed to drive 5 mL Luer lock syringes (Livingstone International), which converted piston position into hydraulic input to the artificial muscles. We used analog control signals generated by feedforward trajectories in MATLAB Simulink (MathWorks, Inc., USA) and output to the motor controllers via a QPIDe Data Acquisition Device (QUANSER, Canada). The ventricular piston pump control signals were designed in the same way as in our previous work [25]. We employed a fourth piston pump to deliver the hydraulic input into both papillary muscles. The magnitude of volume input was controlled by the user during operation to produce the desired valvular performance. The power supply used for the actuators was two series 12 V 138Ah deep cycle batteries (Kings, Australia), which were chosen over mains power due to their ease of implementation and tolerance to voltage dumping as the high‐powered DC motors alternate direction rapidly. Consequently, the current control and actuation system is bulky. Future work could involve optimization of size and weight to increase portability, which was not the focus of this work, but is entirely possible. While we used a PC for prototyping purposes, microcontroller implementation is possible. A mains‐powered power supply is possible too, with voltage clamping circuitry, to reduce the size and weight significantly. Finally, we would like to explore the use of hydraulic gear pumps to replace the relatively bulky linear syringe pumps.

### 5.3 Mock Circulation Loop

The MCL used in this work was constructed in the same way as described in our previous work [25]. However, the increased flow rate produced by our new prototype rendered our small‐bore flow meters meter (Atrato Series Ultrasonic Flow Meter, 0.1–20 L min^−1^, Atrato) too resistive. In lieu of clinical flow meters, we placed our small‐bore ultrasonic flow meters in parallel with large diameter tubes (7/8‐inch ID, 1‐1/8‐inch OD, Tygon PVC soft plastic tubing, McMaster‐Carr), using 3D printed Y‐connectors (). The total flow through the meters and their parallel tubing was calculated using the Darcy‐Weisbach equation. Below, we detail the implementation of this equation for our purposes, where *Q_tot_* is the total flow through both the small‐bore flow meter and the large‐diameter tube, *Q*_1_ is the flow through the small‐bore flow meter, and *Q*_2_ is the flow through the large diameter tube. *v*_1_ and *v*_2_ are the fluid velocities in the small and large tubes respectively. *d*_1_ and *d*_2_ are the small and large tube diameters, respectively.

(1) $$Q_{tot}=Q_{1}+Q_{2}$$

We know that the flow through either the flow meter or the large tube is equal to:

(2) $$Q_{n}=v_{n}\;\left[\pi {\left(\frac{d_{n}}{2}\right)}^{2}\right]$$

where *n* is the tube number 1 or 2.

Rearranging:

(3) $$v_{n}=\frac{Q_{n}}{\pi {\left(\frac{d_{n}}{2}\right)}^{2}}\;$$

We apply the Darcy‐Weisbach equation below:

(4) $$\frac{4f_{1}d_{1}v_{1}^{2}}{2gd_{1}}=\frac{4f_{2}d_{2}v_{2}^{2}}{2gd_{2}}\;$$

Rearranging and simplifying:

(5) $$v_{2}=\sqrt{\frac{l_{1}d_{2}}{d_{1}l_{2}}v_{1}^{2}}\;$$

Substituting Equation (3) into Equation (5) and then into Equation (1), we can express the total flow through both tubes in terms of the measured flow (*Q*_1_) through the flow meter (*l*_1_ = *l*_2_ = 200 mm, *d*_1_ = 6 mm, and *d*_2_ = 22.2 mm):

(6) $$\;Q_{tot}=Q_{1}+\sqrt{\frac{l_{1}d_{2}^{5}}{l_{2}d_{1}^{5}}}Q_{1}=Q_{1}+\sqrt{\frac{200{\left(22.2\right)}^{5}}{200{\left(6\;\right)}^{5}}}Q_{1}=Q_{1}+26.33Q_{1}$$

The variable compliance and resistance elements of the mock loop were manually adjusted to produce the appropriate physiological hemodynamics. The pressure in the LV and aorta was monitored using commercial pressure sensors (40 PC Series Sensor, Honeywell, USA) linked through fluid transmission lines. The flow meter data and this pressure data were recorded and displayed to the user in real‐time via a QPIDe Data Acquisition Device (QUANSER, Canada) and MATLAB Simulink (MathWorks, Inc., USA).

Similar to the control and actuation system, the mock loop was not optimized for portability. Although it is possible to scale this down with close attention to the design of compliance elements (Windkessel chambers) and the minimization of sharp bends in tubing to reduce non‐physiological wave reflections. This could form valuable future work.

### 5.4 Imaging of the Simulator

The echocardiographic imaging of the simulator was performed using a Mindray TE7 Ultrasound System. The probe selection varied between the C5‐1s and the P4‐2s probes, depending on the desired field of view and type of imaging. We utilized the machine's B‐mode imaging to investigate the structural behavior of the simulator, used M‐mode to evaluate the motion of the MV leaflets, and the color Doppler mode to investigate the flow of fluid within the cardiac simulator. Measurements taken from this imaging were compared against clinical criteria to determine the severity of various diseases. A combination of probe placement techniques was used depending on the viewpoint we required. Either the probe was placed directly on the LA membrane, or the left heart simulator was submerged in water, and the probe was simply placed within the water and aimed at the target. We also used the Olympus EVIS X1 CV‐1500 Video System Centre with the Olympus BF‐H1100 Video Bronchoscope to image the MV and the soft robotic catheter operating within the LA above the MV. The bronchoscope was inserted into a port in the LA membrane and manipulated to obtain the desired view.

### 5.5 Design, Fabrication, and Control of the Soft Robotic Catheter

The soft robotic catheter was designed and fabricated in the same way as in our previous work [30], with a 50 mm length and excluding the stabilization mechanism in this preliminary testing. The catheter consisted of three parallel, hydraulic filament artificial muscles that provide omnidirectional freedom (bending and elongation), wrapped in heat‐shrink segments, bonding the muscles together (see Figure S8). The hydraulic actuation for each of the three muscles was provided by three linear stages (Zaber Technologies Inc.) acting as syringe pumps for 3 mL Luer lock syringes (Livingstone International). The motion of these linear stages, and therefore the input volume into the muscles of the catheter, was controlled via Python code. While the data‐driven inverse kinematic model (training described below) was employed to map user commands to muscle input volume, we extracted user commands from the cursor position of a Touch Haptic Device (3D Systems). By initializing the starting cursor position (x, y, z coordinates) and mapping it to the neutral position of the catheter, we were able to take the user's commands relative to this initial position to move the catheter. To enhance the user's experience, we implemented a haptic boundary on the cursor, based on the maximal workspace of the catheter, such that the user was constrained virtually within its workspace. We also wrote Python code to monitor the Touch's cursor position and send it to the inverse kinematic model, which updated the catheter muscle input volume in real time. We also constantly monitored the catheter's muscle pressures in real time and delivered this information to the user as a time‐based plot, using pressure sensors (40 PC Series Sensor, Honeywell, USA) and a QPIDe Data Acquisition Device (QUANSER, Canada) with MATLAB Simulink (MathWorks, Inc., USA).

The data‐driven inverse kinematic model was trained using the multi‐layer perception (MLP) regressor with hidden layers structured as [20, 50, 50, 20], ReLU activation, and Adam solver, with 1200 input/output pairs. We trained using an 80:20 training/validation ratio. We collected the output tip position data using color thresholding of the catheter moving with markers on its base and tip. We recorded the catheter moving with these markers using two cameras and used triangulation to calculate the 3D position of the tip and base of the catheter. The tip position, relative to the base, was correlated with the instantaneous muscle input volume to create input/output pairs. We explored the workspace of the catheter by iterating through stepped permutations of muscle input volume, between the maximum and minimum input volumes. We wrote Python code to iterate through all possible input volumes while simultaneously taking images of the catheter and calculating its 3D base and tip positions.

We also devised a calibration program that takes the workspace tip positions that were measured in the first data collection session and compares these points with 100 random measured tip positions taken whenever the user is about to operate the catheter. This is useful as soft robots are less stable than rigid ones, and their initial state may change over time. By comparing the real tip and base positions for a random set of desired positions, we perform a rotation/ translation operation on the measured positions that minimizes the error relative to those that were desired (Figure S6). This provides us with mapping between expected and actual tip positions.

## Author Contributions

J.J.D., E.N., K.Z., C.C.N., and T.N.D. conceived and developed the concept. J.J.D., E.N., K.Z., and C.C.N. designed and performed experiments. J.J.D., E.N., K.Z., and C.C.N. analyzed the data. J.J.D., T.N.D., and N.H.L supervised and coordinated the project. J.J.D., B.S., A.J., C.C.N, E.N., K.Z., P.T.P., P.P., H.T., J.R.K., H.P.P., C.H., N.H.L., and T.N.D. wrote or revised the manuscript.

## Conflicts of Interest

The authors declare no conflicts of interest.

## Supporting information




**Supporting File 1**: advs74467‐sup‐0001‐SuppMat.docx.

**Supporting file 2**: advs74467‐sup‐0002‐MovieS1.mp4.

**Supporting file 3**: advs74467‐sup‐0003‐MovieS2.mp4.

**Supporting file 4**: advs74467‐sup‐0004‐MovieS3.mp4.

**Supporting file 5**: advs74467‐sup‐0005‐MovieS4.mp4.

**Supporting file 6**: advs74467‐sup‐0006‐MovieS5.mp4.

**Supporting file 7**: advs74467‐sup‐0007‐MovieS6.mp4.

## Data Availability

The data that support the findings of this study are available from the corresponding author upon reasonable request.
