# Supplementary material for: A Soft Robotic Model for Simulating Heart Valve Disease and Cardiac Interventions
Source: Adv Sci (Weinh). 2026 Feb 21;13(27):e16667. doi: 10.1002/advs.202516667 (PMC13170261; doi:10.1002/advs.202516667)
Supplement: Supplementary file 1 — Supporting File 1: advs74467‐sup‐0001‐SuppMat.docx. [file ADVS-13-e16667-s006.docx]

# **Supporting Information**

**A soft robotic model for simulating heart valve disease and cardiac interventions**

James Davies,^1^ Emanuele Nicotra,^1#^ Kefan Zhu,^1#^ Chi Cong Nguyen,^1^ Bibhu Sharma,^1^ Adrienne Ji,^1^ Phuoc Thien Phan,^1^ Jingjing Wan,^1^ Patrick Pruscino, ^1^ Hermione Truong, ^1^ Jelena Rnjak-Kovacina,^1^ Hoang-Phuong Phan,^2^ Christopher Hayward,^3,4^ Nigel Hamilton Lovell,^1,5^ Thanh Nho Do^1,5,^ *

^1^ School of Biomedical Engineering, Faculty of Engineering, UNSW Sydney, NSW 2052, Australia.

^2^School of Mechanical and Manufacturing Engineering, Faculty of Engineering, UNSW Sydney, Sydney, NSW 2052, Australia.

^3^ Department of Cardiology, St Vincent’s Hospital, Sydney, NSW 2010, Australia.

^4^ St Vincent’s Clinical School, Faculty of Medicine, UNSW Sydney, Sydney, NSW 2052, Australia.

^5^ Tyree Institute of Health Engineering (IHealthE), UNSW Sydney, NSW 2052, Australia

**Corresponding author*. Email: tn.do@unsw.edu.au

^#^Equal contribution

## **Investigation into the contraction speed, force-length relationship, and fatigue characteristics of the artificial muscles with respect to native myocardial tissue**

As with any synthetic model, there are differences in the performance of the artificial muscles used in this study and real myocardial tissue. It is therefore necessary to characterize these differences to indicate suitability and potential limitations for their use in simulating cardiac biomechanics and hemodynamics.

It is important to note that the artificial muscles we have chosen, and their hydraulic actuation fluid, effectively consist of only incompressible materials. This means that, not considering fatigue and loading, we do not expect any change in actuator performance with changing actuation frequency. In other words, the volume of the components of the actuator and the volume of the hydraulic liquid are conserved. So, a change in hydraulic input volume produces an equal change in the total volume of the actuator. Since the artificial muscle is radially constrained by a steel coil, this volume change is converted into axial elongation. The high internal actuation pressure will cause some radial coil expansion, contributing to some hysteresis in the input volume – muscle elongation relationship, but this is negligible. In this way, again, without considering fatigue, we believe that there should be no significant damping of muscle strain with increased actuation speed. Therefore, the speed of contraction and relaxation of the muscle is largely restricted by the speed at which we can drive hydraulic fluid in and out of the muscle. This has been a major concern of ours, since the creation of a fast and powerful hydraulic drive system is time- and resource-consuming. While we can achieve actuation at 60 bpm with our current drive system, further work is needed to reach higher heart rates without attenuation of stroke volume.

To demonstrate that this limitation in our current work is a consequence of the current drive system and not of the chosen artificial muscles, we have characterized the contraction speed of a single muscle fiber with a fast and accurate hydraulic drive. We devised a syringe pump using a 1 ml Luer Lock syringe fixed to a precision linear stage (Zaber Technologies Inc.), elongated the muscle fiber, and contracted it as fast as possible with a step input (max acceleration: 11000 mm/s^2, max velocity: 300 mm/s). We recorded the stretch ratio of the muscle fiber loaded with a 50 g mass using a rotary encoder, as seen in Figure S1A. The initial unloaded and contracted muscle fiber length was 95 mm.


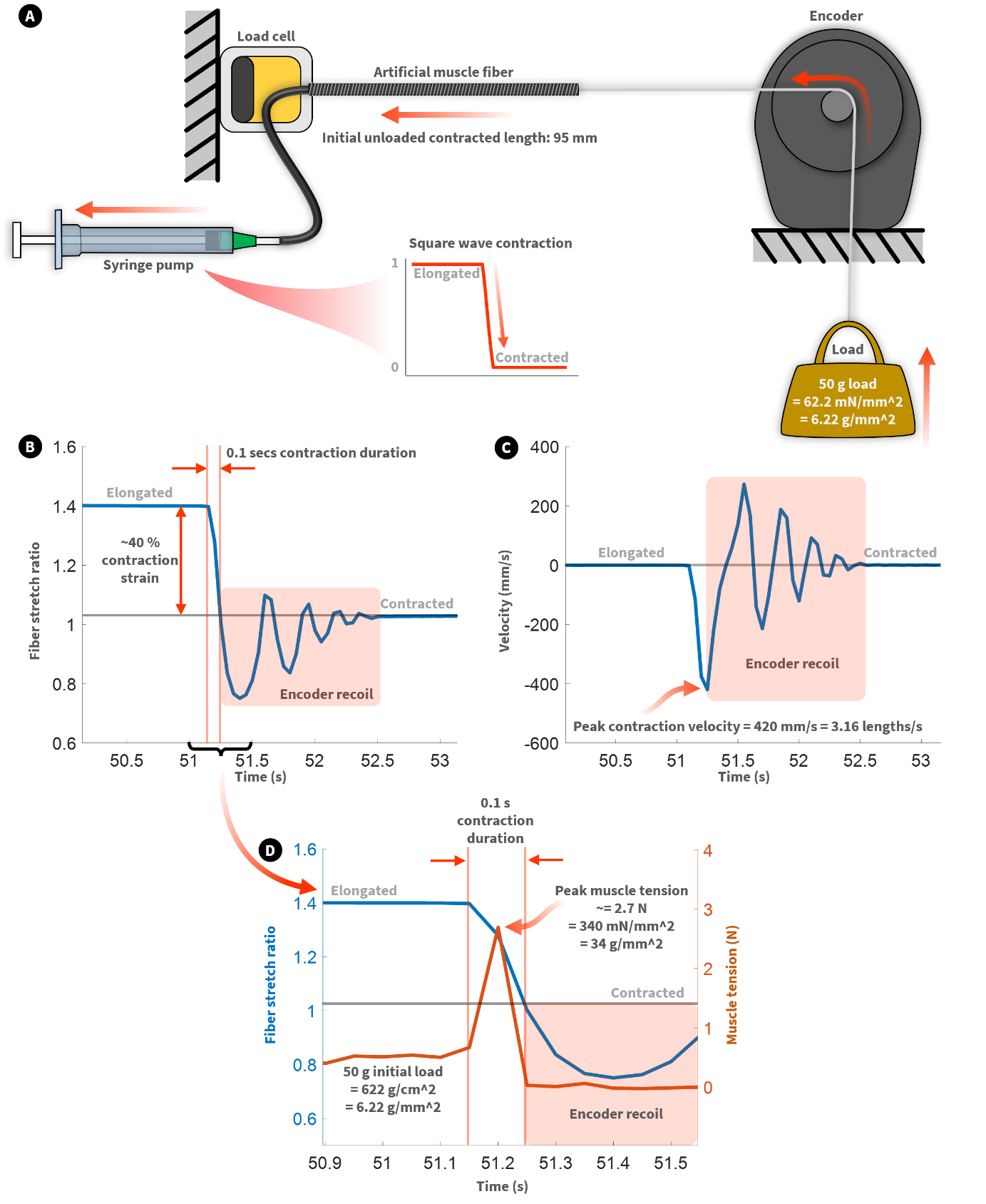


Figure S1. Rapid impulse contraction testing on a single artificial muscle fiber. (A) Experimental setup. (B) Fiber stretch ratio with respect to time. (C) Fiber velocity with respect to time. (D) Fiber stretch ratio and dynamic muscle tension with respect to time.

Since the encoder spindle and the 50 g mass have momentum, there is notable recoil oscillation in the encoder signal after the rapid observed contraction. Ignoring this and observing the contracted stretch ratio after the oscillations have settled, we can see that there was a ~40% contraction strain in a period of 100 ms (Figure S1B), with a peak contraction velocity of 420 mm/s or ~3.2 muscle lengths/s (Figure S1C). 40% contraction strain is approximately equal to the myocardial strain used in this study. Considering the cross-sectional area of the muscle fiber $\left( \pi\left( \frac{3.2mm}{2} \right)^{2} \right)$, the static 50 g preload equates to 6.2 g/mm^2 or 62 mN/mm^2. As seen in Figure S1D, this static preload converts to a 2.7 N afterload upon contraction, equivalent to 34 g/mm^2 or 340 mN/mm^2.

We believe this 100 ms time-to-peak-contraction under supraphysiological load is sufficient for a wide range of heart rates, potentially up to 160 bpm. Regen et al. (*1*) recorded that isovolumic contractions in canine hearts exhibited times-to-peak-contraction ranging from approximately 220 – 101 ms for heart rates ranging from 90 – 160 bpm, respectively. This simple experiment serves as an indicator that high heart rates are possible with our artificial muscles, but that the speed of the hydraulic drive system is the limiting factor when scaling from one isolated muscle fiber to the entire myocardium.

The pre- and after-loads employed in Figure S1 are far greater than those experienced by myocytes in the native heart. Further, we conducted blocked force testing on the same single muscle fiber at increasing blocked stretch ratios and increasing sinusoidal volume inputs into the fiber (Figure S2A). The fiber was initially pressurized to three stretch ratio levels and was then contracted and relaxed at this fixed length using increasing frequency sinusoidal input volume signals. From Figure S2B and C, peak blocked muscle tension was higher when initial blocked fiber stretch ratio was higher. Further, peak blocked muscle tension was not significantly influenced by actuation frequency (ranging from 60 – 120 bpm). At ~40% initial blocked strain, the peak blocked muscle tension was approximately 4.3 N, which is equivalent to 530 mN/mm^2 or 53 g/mm^2. This is higher than that reported mammalian myocardium, ranging from 7 – 11 g/mm^2 (*2*), or 70 – 110 mN/mm^2. While it is known that mammalian myocytes exhibit a positive force-frequency relationship (*3*), our muscle fibers do practically unchanged isometric contraction force with increased contraction frequency. In saying this, since our peak isometric contraction forces are greater than mammalian tissue, it is entirely possible to employ control algorithms to scale down the applied forces to match the native force-frequency relationship. Interestingly, from Figure S2C, we see a positive force-length relationship, mimicking that of mammalian myocytes (*3, 4*). This could be expected as when our artificial muscle is elongated further, more energy is stored in its elastic components, ready to be used in the subsequent contraction. Again, since our artificial muscles are stronger, by cross-sectional area, than native myocardium, we can further tune this force-length relationship with control algorithms.


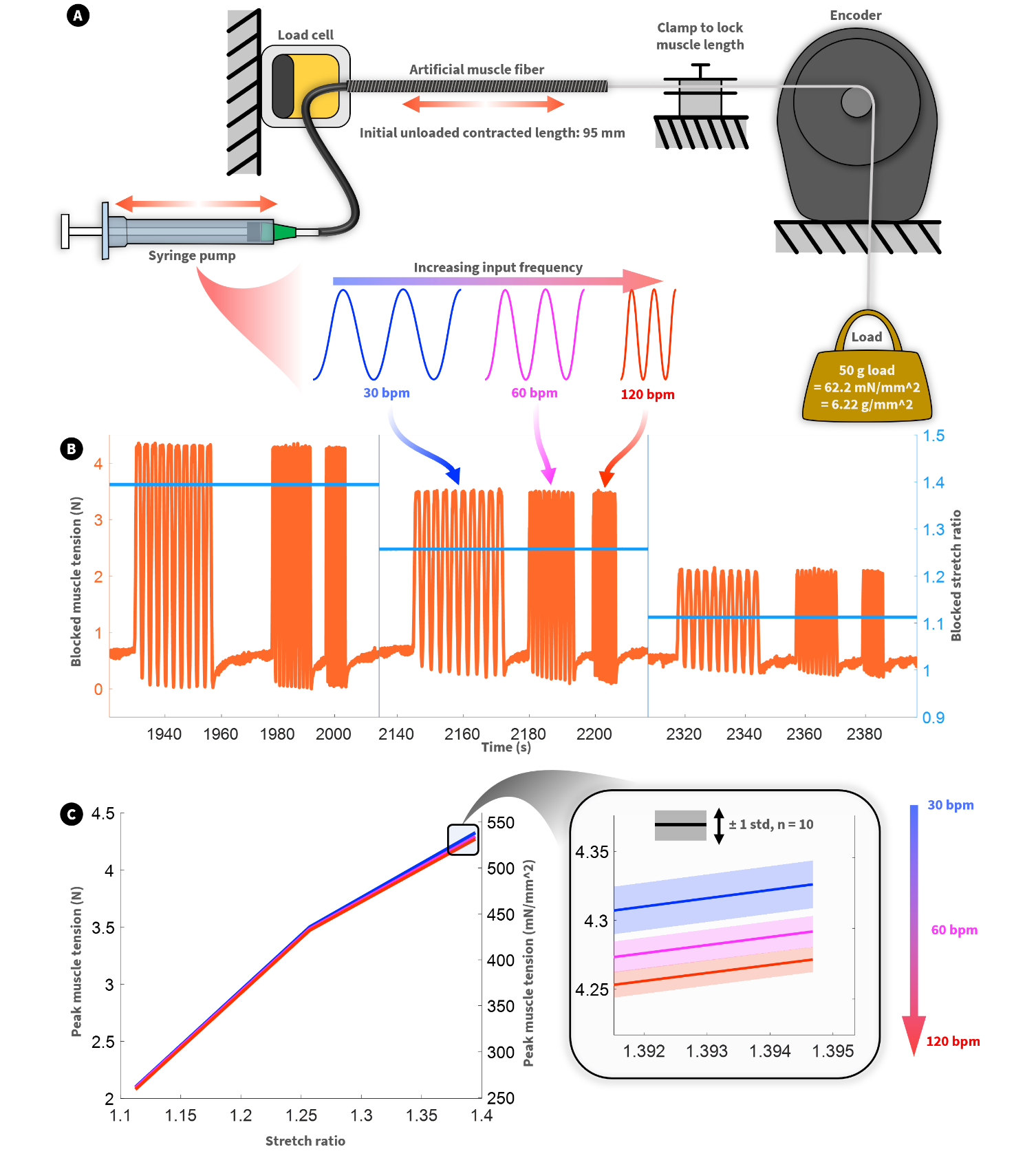


Figure S2. Blocked force testing of a single artificial muscle fiber. (A) Experimental setup. (B) Blocked muscle tension and blocked muscle stretch ratio with respect to time, with increasing frequency contractions. (C) Peak blocked muscle tension developed with respect to stretch ratio and the frequency of the muscle contraction..

We also looked at the effect of increasing load on our artificial muscle fiber in terms of contraction strain and peak contraction velocity (Figure S3). We input a set hydraulic volume into the artificial muscle fiber, loaded it with an increasing static weight, and used a step input to the syringe pump to contract the muscle fiber, all while recording the stretch ratio of the fiber (Figure S3A). Similar to the experiment in Figure S1, this rapid contraction causes recoil oscillations in the encoder as the static load and the encoder’s momentum carry on moving after the muscle has stopped contracting (Figure S3A). Figure S3B reveals the time-domain fiber stretch ratio through contraction for static loads from 100 – 800 g, while Figure S3B shows the fiber velocity for each loading case. Highlighted by Figure S3D, we can see that the muscle’s effective elastic modulus (stretch ratio vs static load) when before and after contraction are different not only in gradient but also in curvature. This produces the interesting contraction strain vs static load relationship in Figure S3E, where increasing load actually increases contraction strain up to a point, before decreasing. However, while the amplitude of the contraction may increase before decreasing, as the load increases, the peak contraction velocity steadily decreases with static load (Figure S3F). This implies that the duration of contraction is longer for the period that contraction strain is increasing with load. Decreasing contraction velocity with increasing load is a known phenomenon in native myocardial tissue (*5*). While our artificial muscles are certainly less sensitive, this invites the opportunity to program increased sensitivity into the artificial myocardium to better simulate the real thing.

It should also be noted that while static load was increased from 100 – 800 g, the dynamic loading during contraction was larger. As seen in Figure S3G, the peak muscle tension developed during each contraction increased non-linearly with static loading. The magnitude of all peak developed muscle tensions far exceeds that of native myocardium, highlighting the power of our artificial muscles when considering their cross-sectional area. The developed peak contraction velocities also exceed that of native myocardium in some reports (*5, 6*), in terms of muscle lengths per second for comparative loads with respect to cross-sectional area.


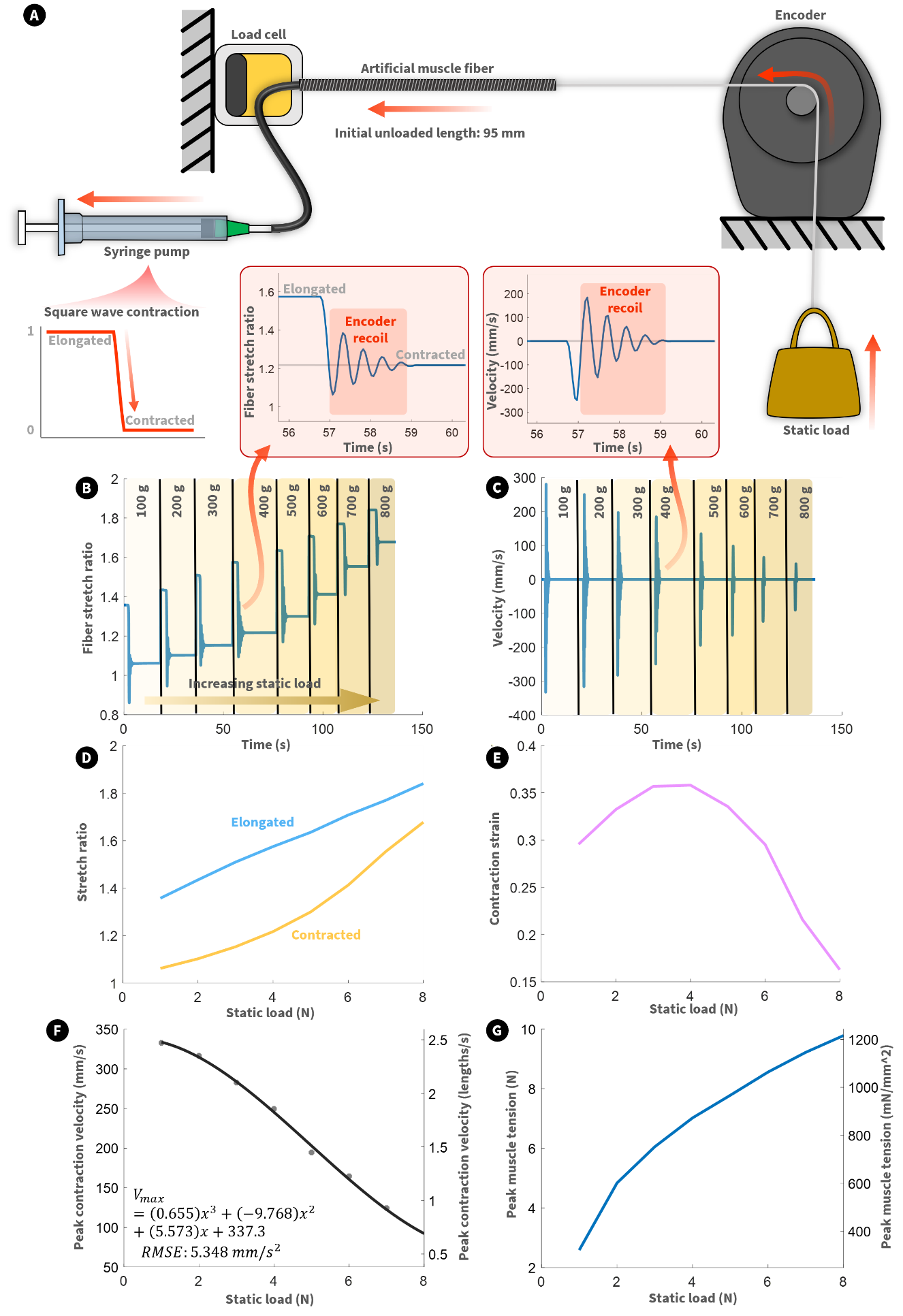


Figure S3. Contraction amplitude and velocity as they vary due to increasing static loading. (A) Experimental setup. (B) Fiber stretch ratio with respect to time and increasing static load from 100 – 800 g. (C) Fiber velocity in the same case as A. (D) Stretch ratio versus static load when the muscle is elongated and contracted. (E) Contraction strain versus static load. (F) Peak contraction velocity vs static load, with fitted curve. (G) Peak muscle tension versus static load.

Summarizing the results above, we believe that the artificial muscles used in this study have great potential for high fidelity cardiac simulation. They are stronger and faster than native myocardium when normalizing with cross-sectional area and length. This invites the opportunity for future work in closed-loop control to allow a simulator to closely mimic the native properties of human myocardium. This supraphysiological power is what allows us to create a myocardial model at less than 100% muscle density while still providing physiological pressure-volume relationships. We have not yet pushed to increase the muscle density in our model, but this may be necessary to achieve optimal performance at higher heart rates.

The bottleneck for the prototype presented in this paper is in fact in the hydraulic pumping system, which is currently not fast or powerful enough to take advantage of the contractile properties of the artificial muscles. Here, we use a hydraulic syringe pump capable of fast and accurate control of a single muscle fiber, not the entire artificial myocardium. Future work will focus on improvements to the current drive system so we can reach higher heart rates and simulate more complicated disease states.

Finally, it is known that increasing strain rate of hyperelastic materials increases the internal stress of the material, even if the strain magnitude is held constant. In our case, that means that increasing the speed of contraction and relaxation will increase the stress experienced by the hyperelastic rubber tube in our artificial muscles. It is therefore expected that fatigue will build up faster in the muscles when we operate at higher heart rates. However, temperature effectively decreases the stress-strain relationship. This may be an important consideration since the artificial muscles do heat up internally with increased contraction/relaxation speeds. But it is expected that extended periods under high stress will cause progressive compaction or increases in density over time, which will result in a reduction of elongation with respect to input volume over time. As seen in Figure S4, we conducted fatigue testing on the same muscle fiber as the above experiments, with a 100 g static load, equivalent to 12 g/mm^2 or 120 nM/mm^2. We applied a continuous sinusoidal volume input with a 0.5 Hz frequency with approximately 50% strain, equivalent to 120 bpm in heart rate (Figure S4A). Figure S4B shows the shrinkage in stretch ratio amplitude was 5% over 4275 cycles, but this stabilized rapidly, and if one considers the shrinkage after the initial stabilization, this reduces to 0.7%. The reason we stopped at 4275 cycles was not due to muscle failure, but rather due to the hydraulic syringe’s plunger wearing out (Figure S4A). Clearly, more durable options need to be considered as these Luer Lock syringes are not built for such applications. That said, after the initial decay in stretch ratio amplitude, the muscle’s performance under load is very stable. Although more testing is necessary to comment on truly long-term simulation.


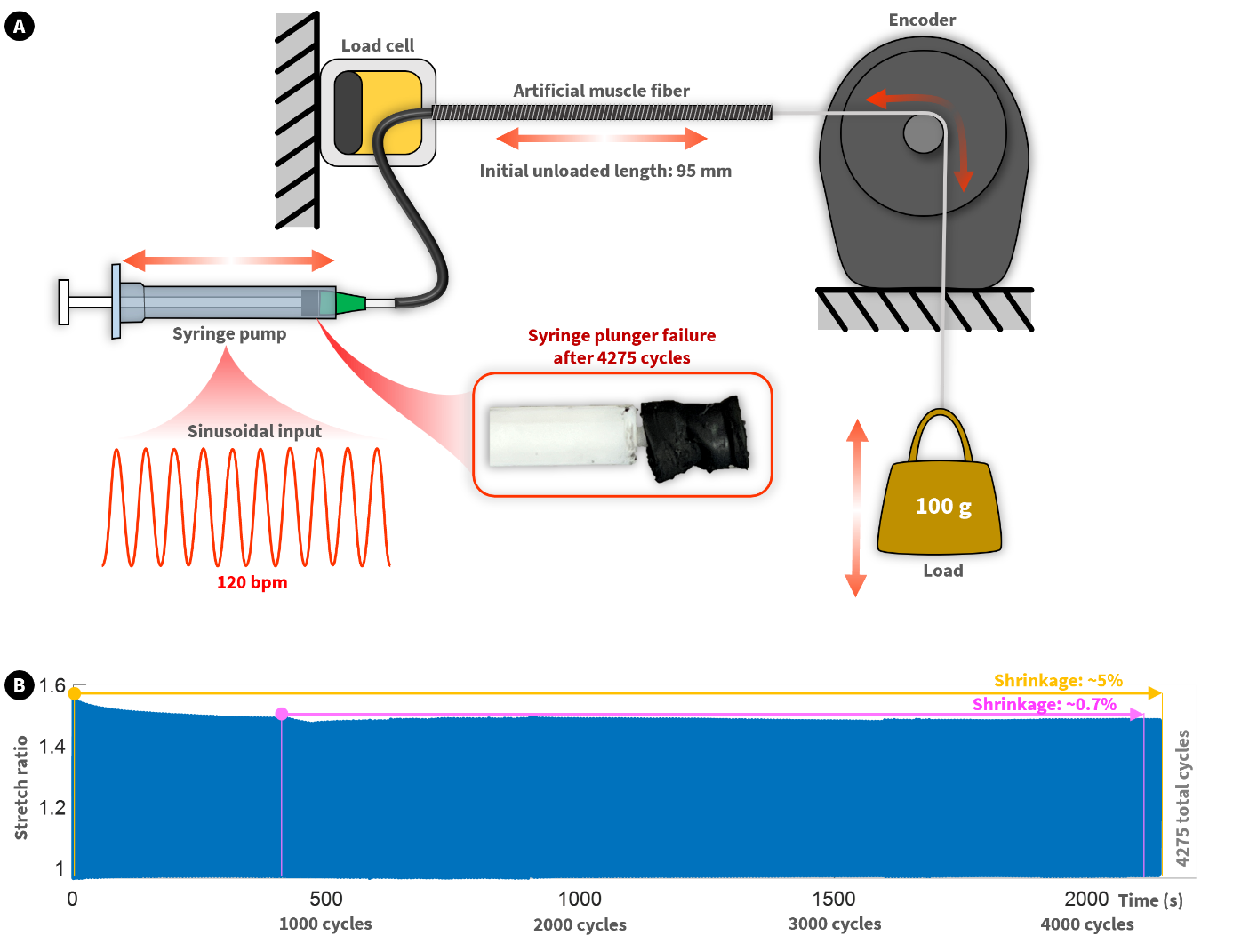


Figure S4. Fatigue testing of a single muscle fiber under 100 g static loading. (A) Experimental setup. (B) Fiber stretch ratio over 4275 continuous elongation/contraction cycles at 0.5 Hz or 120 bpm, with stretch ratio shrinkage reported for the entire testing period and the period after initial stabilization.

References

1. D. M. Regen, W. C. Howe, J. T. Peterson, W. C. Little, Characteristics of single isovolumic left-ventricular pressure waves of dog hearts in situ. *Heart and Vessels* **8**, 136-148 (1993).

2. E. H. Sonnenblick, W. W. Parmley, R. A. BUCCINO, J. F. SPANN, Maximum force development in cardiac muscle. *Nature* **219**, 1056-1058 (1968).

3. P. M. Janssen, Myocardial contraction-relaxation coupling. *American Journal of Physiology-Heart and Circulatory Physiology* **299**, H1741-H1749 (2010).

4. G. Iribe, M. Helmes, P. Kohl, Force-length relations in isolated intact cardiomyocytes subjected to dynamic changes in mechanical load. *American Journal of Physiology-Heart and Circulatory Physiology* **292**, H1487-H1497 (2007).

5. E. H. Sonnenblick, Instantaneous force-velocity-length determinants in the contraction of heart muscle. *Circulation Research* **16**, 441-451 (1965).

6. D. L. Fry, D. M. Griggs, J. C. Greenfield, Myocardial mechanics: Tension-velocity-length relationships of heart muscle. *Circulation Research* **14**, 73-85 (1964).


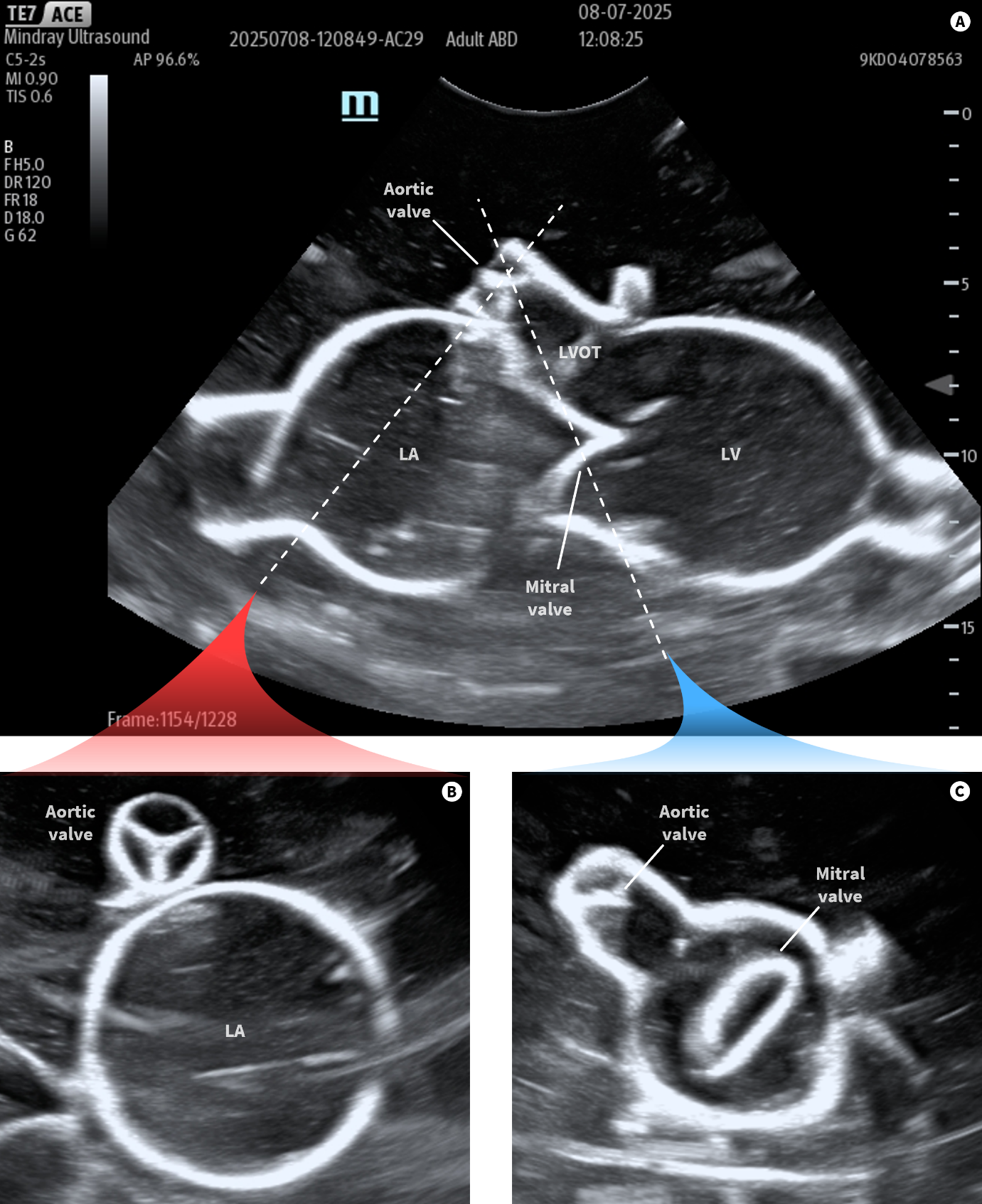


Figure S5. Design iteration for cardiac membranes for better ultrasound compatibility. (A) Longitudinal view showing major cardiac features. (B) Radial view showing aortic valve leaflets and LA. (C) Radial view showing both aortic and mitral valve.


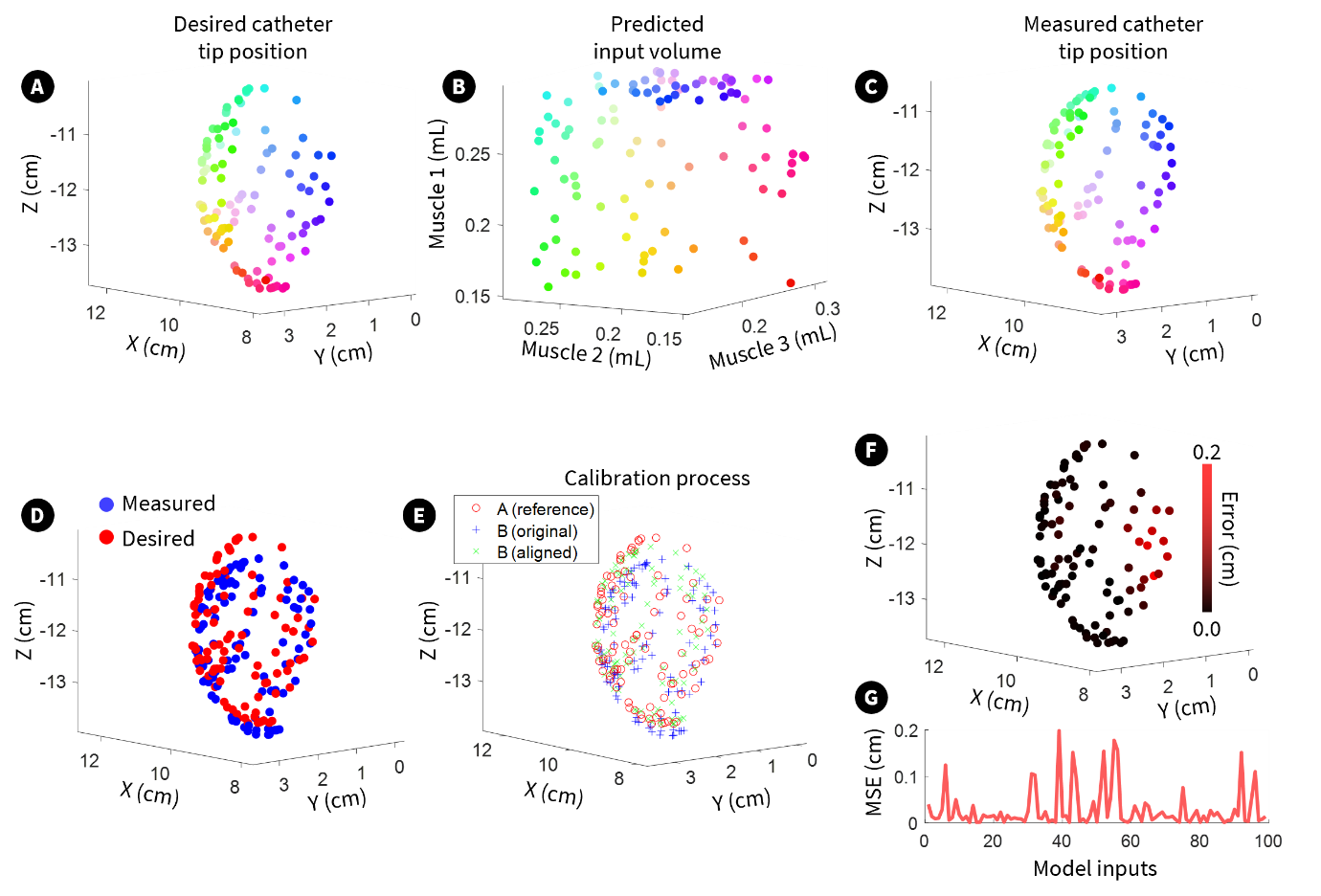


Figure S6. Calibration process and performance of the data driven inverse kinematic model. (A) The set of desired catheter tip positions input into the model. (B) The predicted muscle input volumes calculated by the inverse model. (C) The output measured tip positions corresponding to the predicted input volumes. (D) The desired and measured tip positions relative to one another. (E) The calibration of the two sets of data with a workspace rotation and translation to minimize error. (F) The desired tip positions from the calibration set with color plotting corresponding to the error measured from the physical catheter. (G) Mean square error (MSE) from input to measured output tip position across 100 sampled goals.


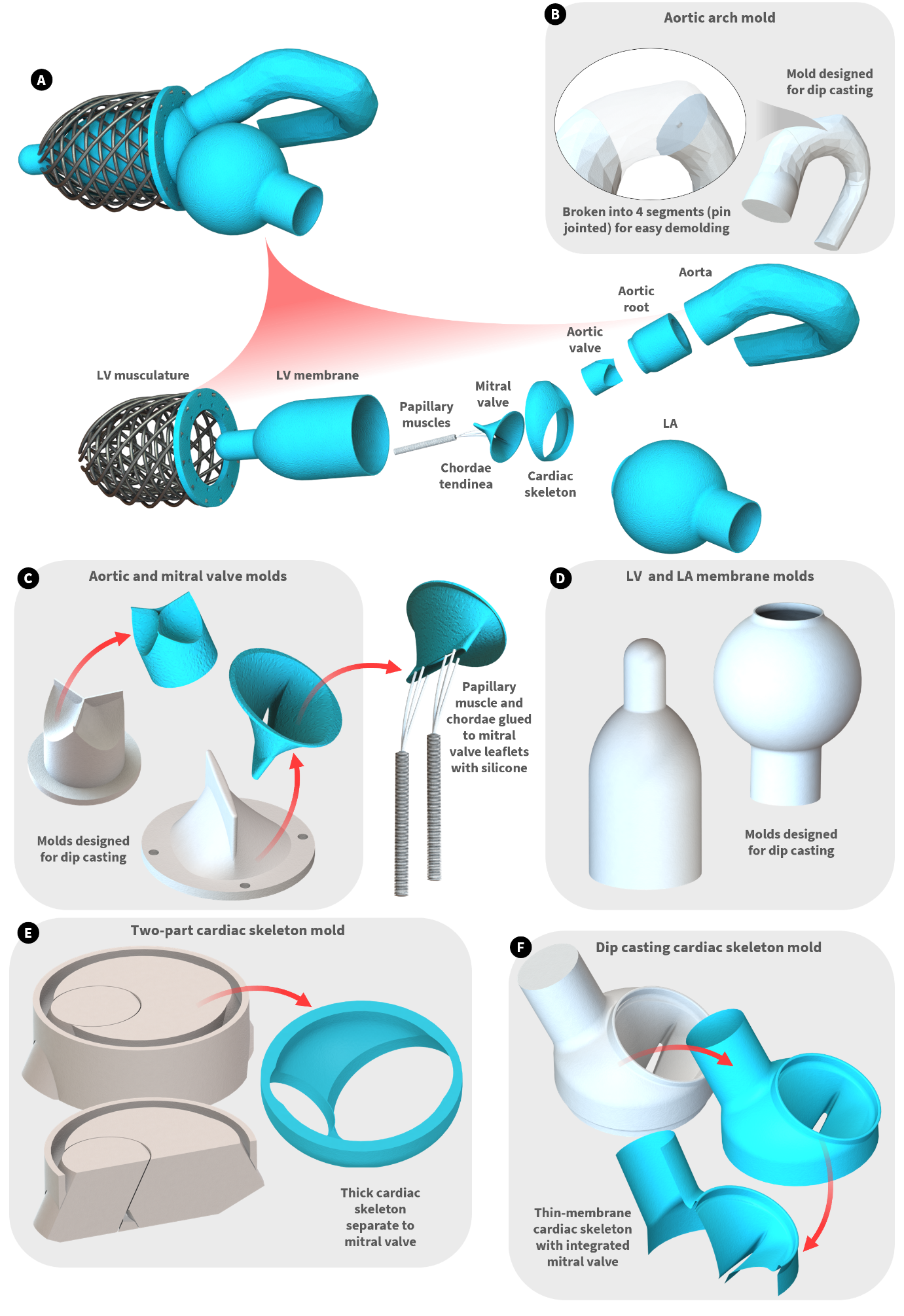


Figure S7. Design and fabrication of the left heart simulator. (A) Exploded view with all components. (B) 3D printed mold for the aortic arch. (C) 3D printed molds for the aortic/mitral valves. (D) 3D printed molds for the LV and LA membranes. (E) 3D printed, 2-part mold for the cardiac skeleton. (F) A 3D printed dip casting mold for the cardiac skeleton.


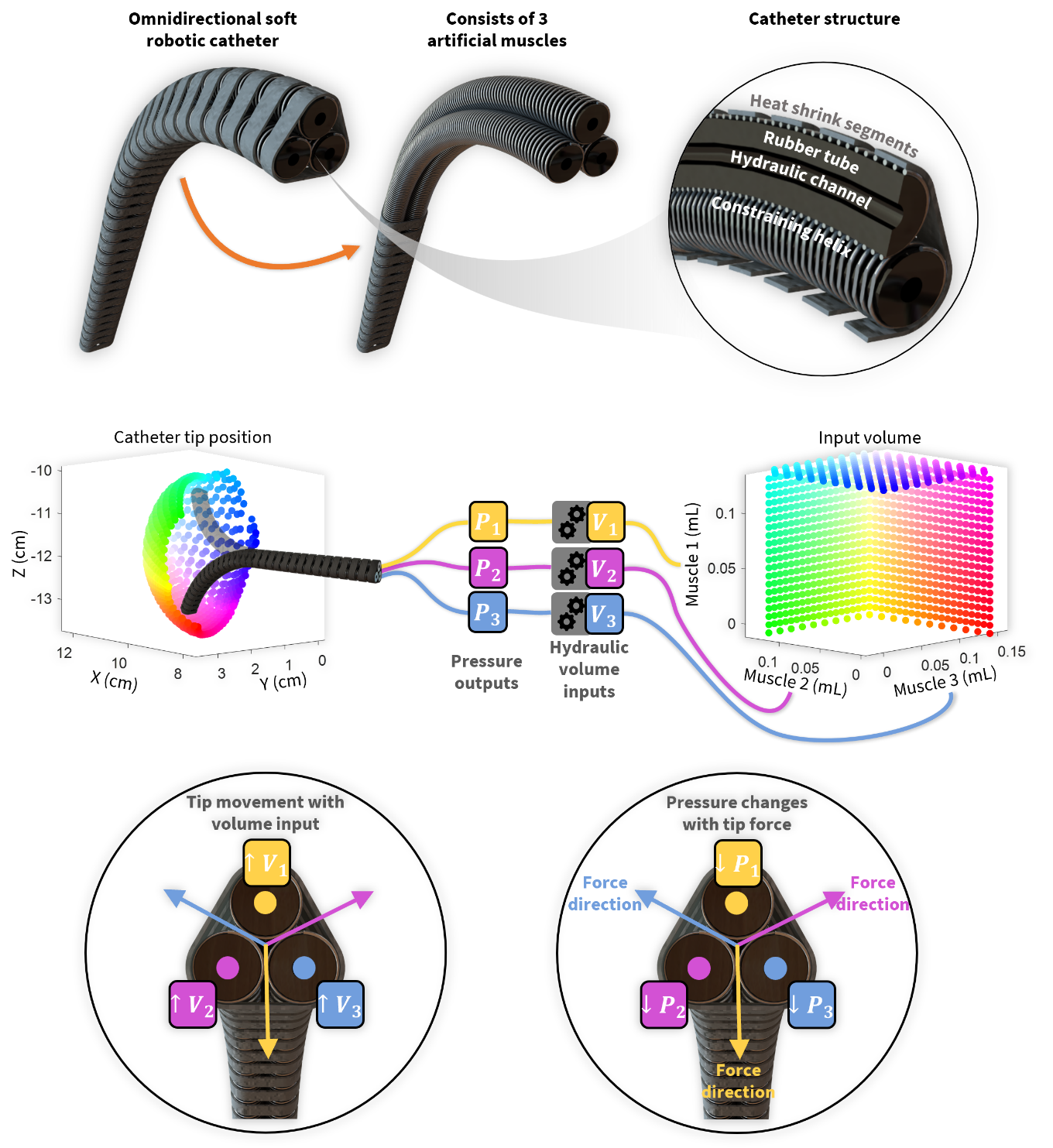


Figure S8. Structure and working principle of the soft robotic catheter. It consists of three hydraulic filament artificial muscles aligned in parallel and stabilized to each other with heat shrink segments. The heat shrink is segmented to allow for bending and elongation. The artificial muscles themselves consist of a helical fiber radially constricting a rubber tube. Volume input into these muscles results in elongation. When combined in the catheter, individual muscles will cause bending, while combined input of all three muscles allows elongation.


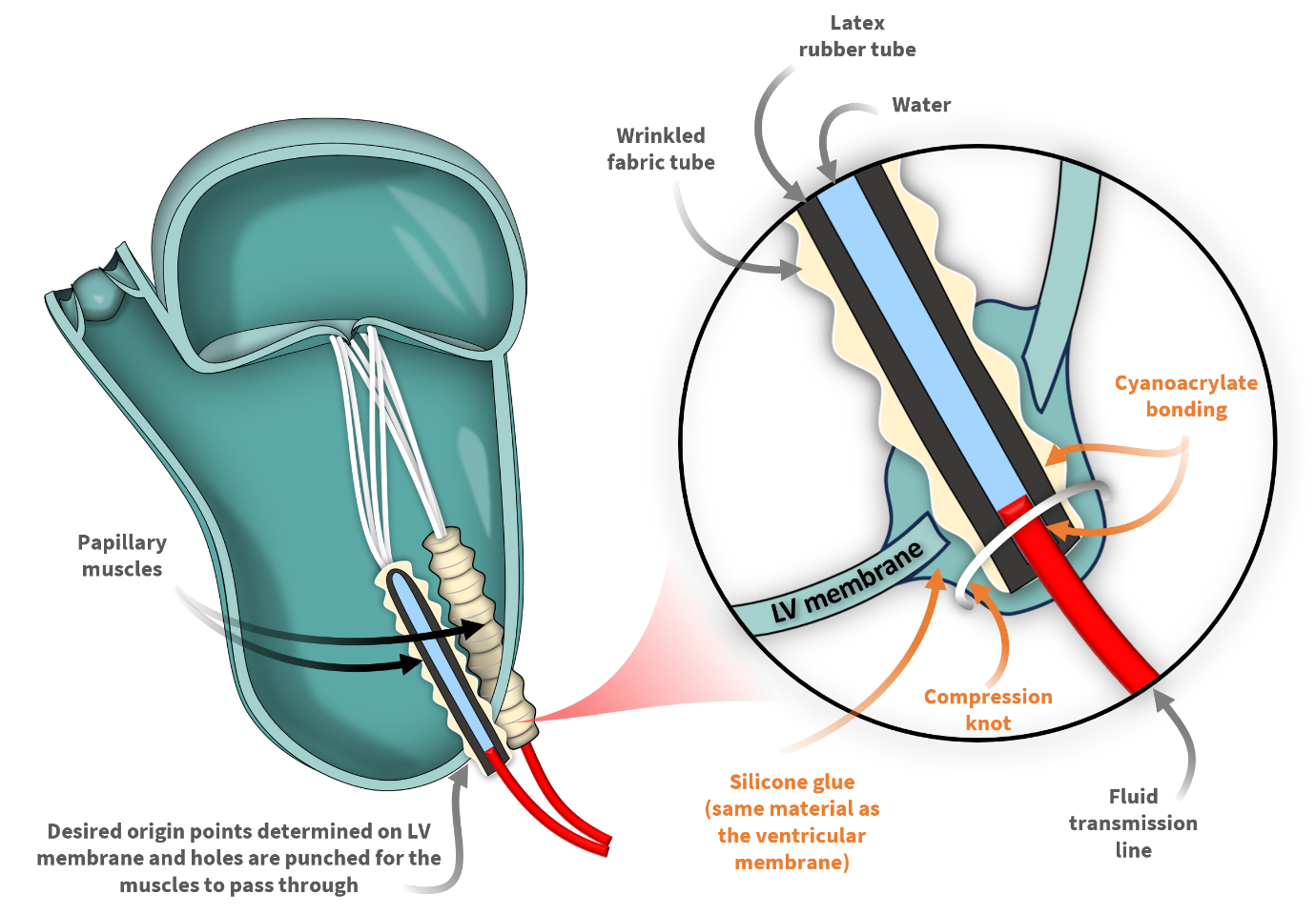


Figure S9. Detail on the integration of the papillary muscles into the left ventricular membrane.


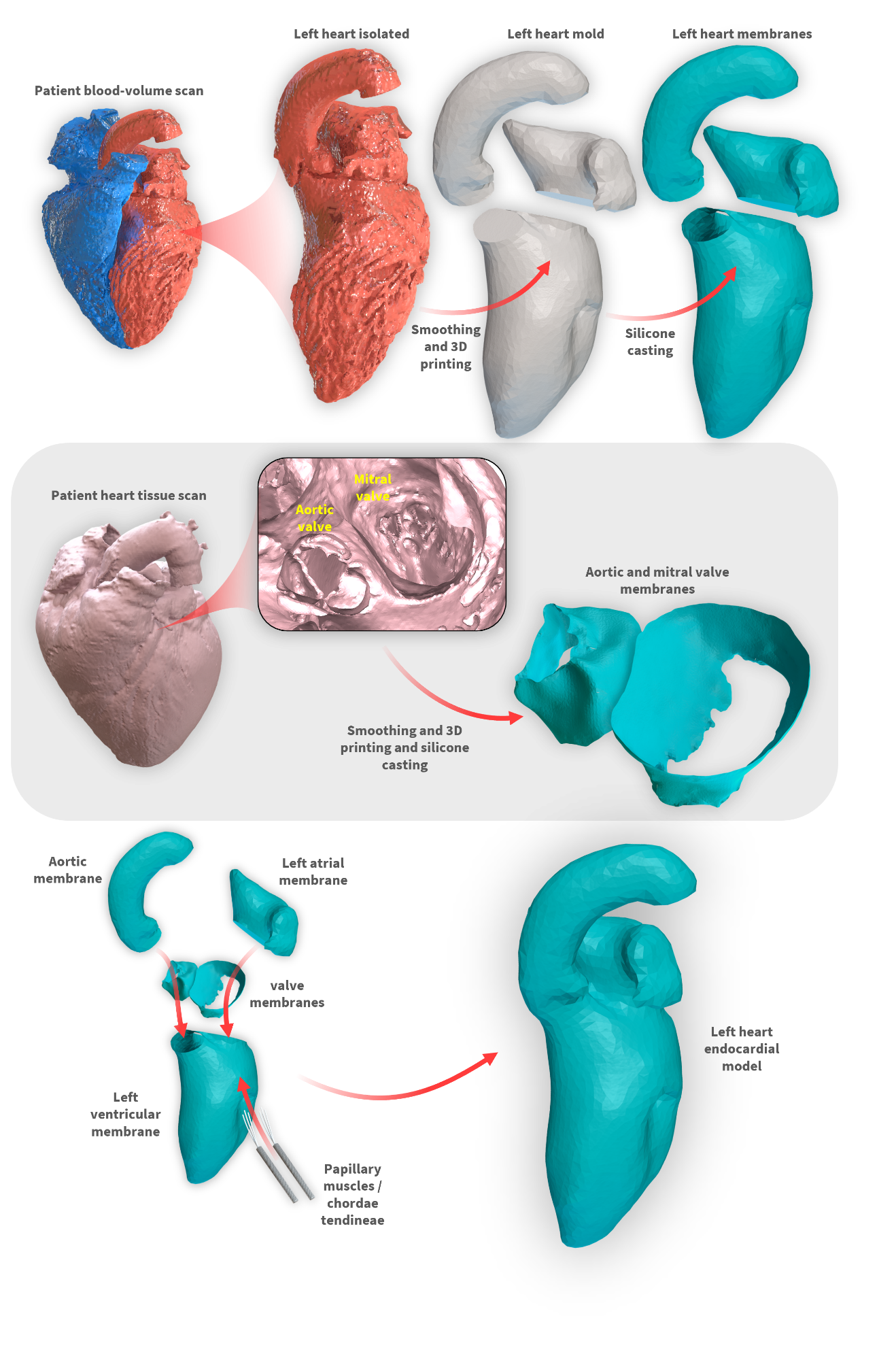


Figure S10. Envisioned future workflow to extract patient-specific geometry to construct patient-specific cardiac membranes.


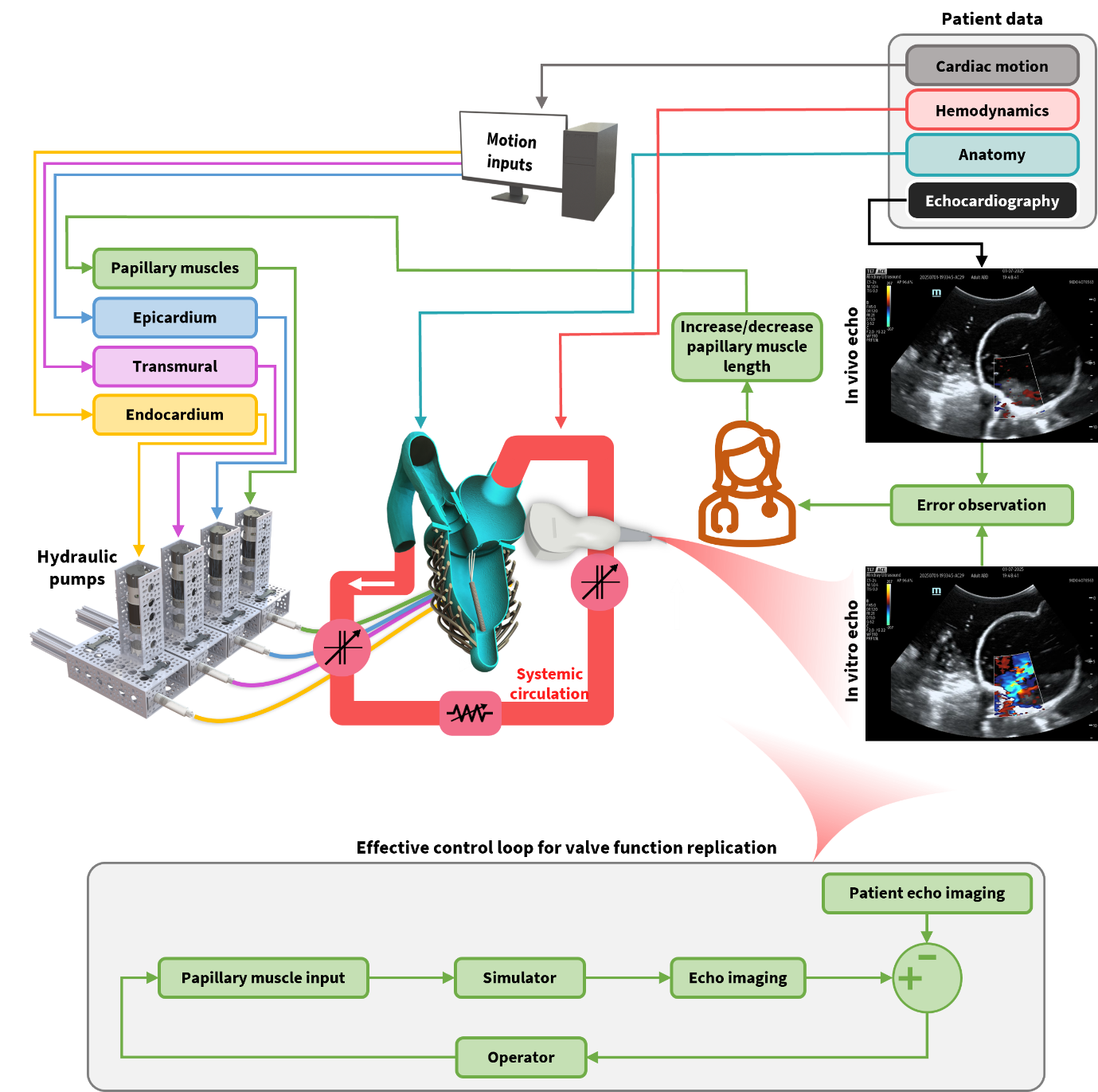


Figure S11. Envisioned workflow and control diagram for design, fabrication, and control of the simulator, including the human-in-the-loop control over papillary muscle function.
